# Uncovering the Genetic History of the Present-Day Greenlandic Population

**DOI:** 10.1016/j.ajhg.2014.11.012

**Published:** 2014-12-31

**Authors:** Ida Moltke, Matteo Fumagalli, Thorfinn S. Korneliussen, Jacob E. Crawford, Peter Bjerregaard, Marit E. Jørgensen, Niels Grarup, Hans Christian Gulløv, Allan Linneberg, Oluf Pedersen, Torben Hansen, Rasmus Nielsen, Anders Albrechtsen

**Affiliations:** 1 The Bioinformatics Centre, Department of Biology, University of Copenhagen, 2200 Copenhagen, Denmark; 2 Department of Human Genetics, University of Chicago, Chicago, IL 60637, USA; 3 Department of Integrative Biology, University of California, Berkeley, Berkeley, CA 94720, USA; 4 UCL Genetics Institute, Department of Genetics, Evolution, and Environment, University College London, London WC1E 6BT, UK; 5 Centre for GeoGenetics, Natural History Museum of Denmark, University of Copenhagen, 1350 Copenhagen, Denmark; 6 National Institute of Public Health, University of Southern Denmark, 1353 Copenhagen, Denmark; 7 Steno Diabetes Center, 2820 Gentofte, Denmark; 8 The Novo Nordisk Foundation Center for Basic Metabolic Research, Section of Metabolic Genetics, Faculty of Health and Medical Sciences, University of Copenhagen, 2100 Copenhagen, Denmark; 9 Arctic Centre at the Ethnographic Collections, National Museum of Denmark, 1220 Copenhagen, Denmark; 10 Research Centre for Prevention and Health, Glostrup University Hospital, 2600 Glostrup, Denmark; 11 Department of Clinical Experimental Research, Glostrup University Hospital, 2600 Glostrup, Denmark; 12 Department of Clinical Medicine, Faculty of Health and Medical Sciences, University of Copenhagen, 2200 Copenhagen, Denmark; 13 Faculty of Health Sciences, University of Southern Denmark, 5000 Odense, Denmark; 14 Department of Statistics, University of California, Berkeley, Berkeley, CA 94720, USA

## Abstract

Because of past limitations in samples and genotyping technologies, important questions about the history of the present-day Greenlandic population remain unanswered. In an effort to answer these questions and in general investigate the genetic history of the Greenlandic population, we analyzed ∼200,000 SNPs from more than 10% of the adult Greenlandic population (n = 4,674). We found that recent gene flow from Europe has had a substantial impact on the population: more than 80% of the Greenlanders have some European ancestry (on average ∼25% of their genome). However, we also found that the amount of recent European gene flow varies across Greenland and is far smaller in the more historically isolated areas in the north and east and in the small villages in the south. Furthermore, we found that there is substantial population structure in the Inuit genetic component of the Greenlanders and that individuals from the east, west, and north can be distinguished from each other. Moreover, the genetic differences in the Inuit ancestry are consistent with a single colonization wave of the island from north to west to south to east. Although it has been speculated that there has been historical admixture between the Norse Vikings who lived in Greenland for a limited period ∼600–1,000 years ago and the Inuit, we found no evidence supporting this hypothesis. Similarly, we found no evidence supporting a previously hypothesized admixture event between the Inuit in East Greenland and the Dorset people, who lived in Greenland before the Inuit.

## Introduction

With its more than 2,150,000 km^2^, Greenland is the largest island in the world. However, because of its cold climate and remote location, it has historically been only sparsely populated, and the size of its present-day population is only about 57,000 individuals.

Archeological evidence indicates that Greenland was colonized several times when people from northeastern Canada entered into the northwestern part of the island.^1^ The Paleo-Eskimos of the Independence I culture and of the Saqqaq culture were the first to populate the island ca. 2500 BC. The former group settled in North and Northeast Greenland, and the latter settled in West and Southeast Greenland. Around 800 BC, a new group of Paleo-Eskimos representing the Dorset culture arrived. In North and Northeast Greenland, this culture is labeled Independence II. From approximately 1 AD to the 8^th^ century AD, no human activity was documented anywhere on the island. Then Paleo-Eskimos of the Late Dorset culture settled in the Thule district in North Greenland, where they lived until ca. 1300 AD. The Neo-Eskimos, i.e., Inuit of the Thule culture, arrived in the same area from Alaska through Canada in the 12^th^ century, and archaeology has provided evidence of the coexistence of the two groups in this area.^2^, ^3^ In 985, the Norse Vikings settled in the southern part of West Greenland, where they remained until about 1450 AD. Archaeology has provided substantial evidence of contact between Norse, Late Dorset, and Inuit pioneers.^2^, ^3^ These interactions did not necessarily take place close to the Norse settlements but could have taken place anywhere in West Greenland.^2^ From the 14^th^ century onward, the Inuit settled in West and Southeast Greenland. They also traveled north around the country and settled in Northeast Greenland for four centuries, and several archeological studies have suggested that a gradual migration south into Southeast Greenland originated there.^4^, ^5^ In the 17^th^ century, the Inughuit Polar Eskimos (new Inuit people from the central Canadian Arctic) settled in the Thule district. By this time, the last group of the Inuit pioneers had left Thule and settled in Upernavik, the northernmost part of West Greenland.^3^ In 1721, the Norwegian priest Hans Egede initiated a period during which Greenland was a Danish colony, which lasted until 1953 and ended formally in 1979, when Denmark granted home rule to Greenland.

Genetic studies have shown that many modern Greenlanders have a substantial amount of European ancestry^6^, ^7^, ^8^, ^9^ inherited mainly from male Europeans.^6^ Furthermore, a large genetic study based on DNA from historic samples from different arctic cultures including Saqqaq, Dorset, and Thule, as well as two whole genomes from present-day Greenlanders, was recently published.^10^ This study provided genetic evidence showing that modern-day Inuit in Greenland are direct descendants of the first Inuit pioneers of the Thule culture. However, despite these advances, several central questions regarding the history of the Greenlandic population remain unanswered, mainly because of a lack of genome-wide data from a large sample of Greenlandic individuals. For example, it is still unknown whether the Norse Vikings are among the ancestors of the Greenlanders. No physical or dental anthropological evidence has been found in support of admixture between the Inuit and the Norse,^11^ but the two populations were in Greenland at the same time, and sagas, Papal briefs,^12^ and archeological findings suggest that contact took place.^2^ A few attempts were made to answer this question with genetics, but all were unsuccessful; part of the reason is that the Norse Vikings came from the same or similar geographical regions as the later European colonizers, making it difficult to answer this question by inferring the source country of the European ancestors of the Greenlanders.^6^ Moreover, any potential genetic contribution from the Norse Vikings is most likely small and would have left a very limited genetic footprint, making the amount of genetic data used in previous studies insufficient.

Another unanswered question is which migration route or routes the first Inuit pioneers used when settling Greenland. About 80% of the island is covered by an ice sheet, making it impossible to settle and access noncoastal areas. As a consequence, present-day Greenlanders live in villages along the coast; the north and northeast coasts remain unoccupied, and the east coast has only a few remote villages that are difficult to reach. Several migration routes have been hypothesized. The possibility of a single wave of migration starting in North Greenland and moving south down the west coast and from there reaching East Greenland is mentioned by Helgason et al.^13^ In contrast, on the basis of archaeological and linguistic evidence, Gulløv^14^ argues that there were two major migration routes, both starting in Northwest Greenland. One wave of migration expanded down the west coast, entered Southwest Greenland after the depopulation of the Norse settlements, and ended on the east coast of Greenland. The other migration wave expanded along the northern coast to ultimately reach the east coast, where it encountered the descendants of the western expansion wave in the 16^th^ century. Helgason et al.^13^ also argue in favor of this latter scenario and further hypothesize, on the basis of mtDNA analyses, that Thule Inuit encountered and interbred with existing Dorset culture individuals both in Canada and on the east coast of Greenland. They argue that Inuit in East Greenland and North Greenland share mtDNA haplotypes and are differentiated from South and West Greenlanders; this would not be expected had only the first of the two migration waves taken place and had there not been any interbreeding with the Dorset on the east coast. Some linguistic evidence also supports a connection between East and North Greenlanders with similar dialects in Upernavik in Northwest Greenland and East Greenland.^5^, ^15^

In line with Helgason et al.’s hypothesis of admixture between the Inuit and the Dorset, the Inuit crossed a region occupied by the Dorset people when they spread eastward from Alaska around 1200 AD, and the Dorset are mentioned in the legends of the Inuit as a distinct people called Tunit.^16^ Hence, the Inuit and the Dorset most likely encountered each other at some point and might have interbred. Furthermore, the previously mentioned study based on ancient DNA showed that it is possible that some admixture took place long before the Inuit arrived in Greenland.^10^ With regard to admixture in Greenland, some anthropologists have suggested that there might have been pockets of surviving Dorset people in Greenland when the Inuit arrived, although McGhee^17^ argues that the available archeological evidence does not support this hypothesis. Therefore, the validity of Helgason’s hypothesis regarding interbreeding of Inuit and Dorset people in East Greenland remains an open question.

Finally, the genetic structure within the present-day Greenlandic population remains poorly described. In particular, the amount of European admixture has yet to be thoroughly quantified, both at a population level and at a regional level. Thus, it is not known whether European ancestry is equally distributed across Greenland or largely restricted to easily accessible towns on the west coast of Greenland, as might be expected given that European immigrants have concentrated in these localities historically.

To answer the above questions and thereby reconstruct important parts of the history of the present-day Greenlandic population, we analyzed almost 200,000 genetic markers from a large population sample consisting of more than 10% of the adult Greenlandic population.

## Material and Methods

### SNP Chip Data Sets

**Figure 1:**
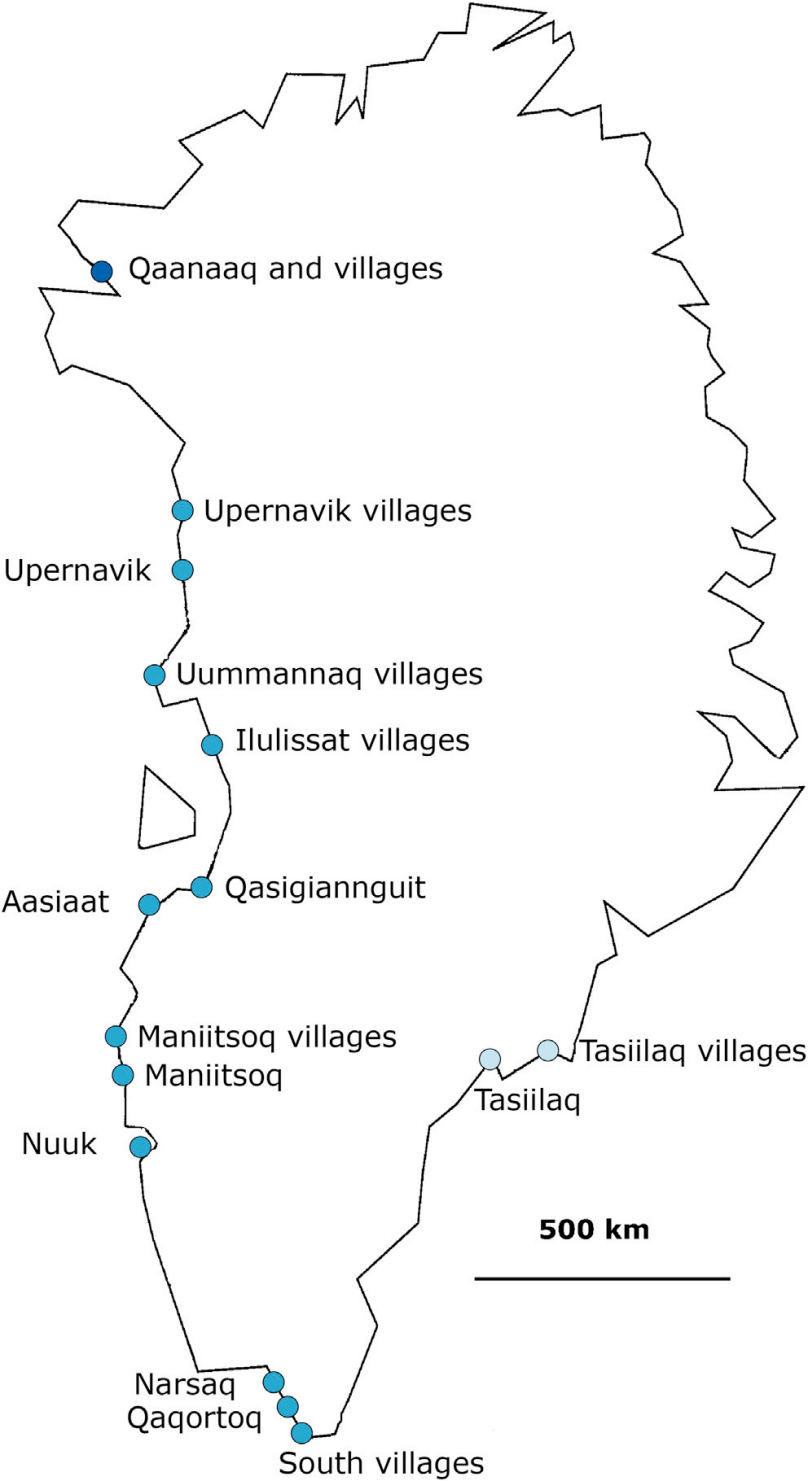
Sampling Locations in Greenland In this map of Greenland, dots illustrate the towns and villages where the Greenlandic participants are from. Note that for some of the locations, the dot represents several small villages up to 100 km apart. The color scheme reflects the geographical division of Greenland into north (dark blue), west (including south, medium blue), and east (light blue).

The analyses in this study were based on genetic data from 4,674 Greenlandic participants from three different cohorts. Of these individuals, 4,127 were participants of the Inuit Health in Transition (IHIT) cohort^18^ and/or the general population health survey (B99)^19^ from locations all over Greenland (Figure 1). The remaining 547 were from a cohort^19^ consisting of individuals who have Greenlandic ancestry and live in Denmark. In addition to data from the Greenlandic individuals, genetic data from 50 Danish individuals from the Inter99 cohort^20^ were included to represent Europeans. All but the 547 individuals from the cohort of Greenlanders living in Denmark were genotyped as a part of a recent disease study^8^ with the Illumina CardioMetaboChip^21^ (MetaboChip), which consists of 196,224 SNPs. About half of these SNPs are rare. We used the same chip to genotype the participants from the cohort of Greenlanders living in Denmark for this study. Additionally, Illumina genotyped the four original HapMap populations^22^ on the MetaboChip, and we used these genotypes to facilitate comparison between the Greenlandic population and other populations. From these data, we made two data sets, on which almost all analyses presented here are based.

1. The full data set consisting of data from all 4,674 Greenlandic and all 50 Danish individuals.
2. A restricted data set consisting of a subset of the Greenlandic individuals who are not closely related, who have no recent European ancestry (<5% estimated European ancestry), and who are not recent migrants within Greenland. When this restricted data set was used in analyses, genetic data from this restricted subset of Greenlandic individuals were either combined with data from the 50 Danish individuals or data from 60 unrelated individuals with European ancestry, 44 unrelated Han Chinese individuals, 45 unrelated Japanese individuals, and 59 unrelated Yoruba individuals, all from HapMap.

Below is a detailed description of each of these data sets and what filters were applied to them.

#### The Full Data Set

**Figure 2:**
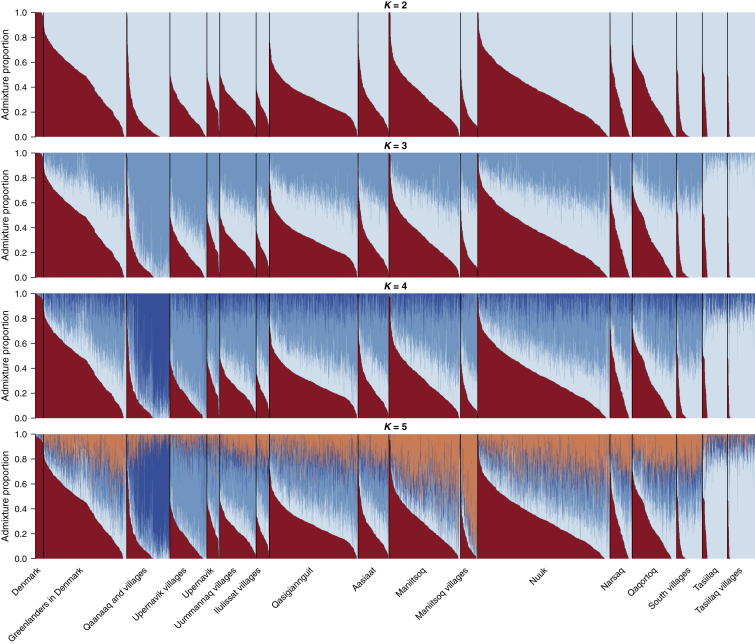
Estimated Admixture Proportions for Individuals from Different Locations in Greenland Admixture proportions for all Greenlandic and Danish individuals in this study (the full data set) were estimated with ADMIXTURE under the assumption of different numbers of ancestral populations (*K* = 2–5). When another component was added (*K* = 6), the additional component did not correlate with location (data not shown). For each *K*, the results are ordered according to where the individuals are from. The color scheme for *K* = 4 is the same as in Figure 1.

To make this data set, we combined (1) genetic data from all genotyped participants of the IHIT cohort and the general population health survey (B99) from 15 locations in Greenland (Figure 1), (2) genetic data from all genotyped participants of the cohort of Greenlanders living in Denmark, and (3) genetic data from 50 Danish individuals from the Inter99 cohort. After merging the data sets, we removed all individuals who appeared in more than one cohort so that each individual was represented only once. We also removed all individuals with more than 2% missing genotypes among the SNPs with a minor allele frequency (MAF) above 1% and all individuals with misspecified or missing gender information. This left us with 4,127 Greenlandic individuals living in Greenland, 547 Greenlandic individuals living in Denmark, and 50 Danish individuals for a total of 4,724 individuals. From this data set we removed all SNPs with a MAF below 5% and/or more than 1% missingness, which left us with data from 92,362 SNPs, of which 92,151 were autosomal. The nonautosomal SNPs were only included in analyses where this is specifically stated.

#### The Full Data Set without LD

Several of our analyses were based on Greenlandic allele frequencies corrected for European admixture. To create the data set we used to estimate these allele frequencies, we extracted the MetaboChip data from the 4,127 Greenlandic individuals living in Greenland from the full data set and then filtered out SNPs in strong linkage disequilibrium (LD) by retaining only one SNP for each pair of SNPs showing an *r*^2^ value greater than 0.3 in windows of 50 SNPs by using a step size of five SNPs. This left us with 63,911 SNPs.

#### The Restricted Greenlandic Data Set Combined with Danish Samples

On the basis of admixture proportions estimated with the autosomal SNPs from the full data set under the assumption of two ancestral populations (*K* = 2), we identified and removed all individuals with an estimated European ancestry proportion above 5%. From the remaining individuals, we extracted individuals from Qaanaaq, Upernavik villages, South Greenland villages (South villages), Tasiilaq villages, and Tasiilaq, which were the only Greenlandic sampling locations with more than 15 individuals left. This left us with 584 Greenlandic individuals with no or very little European ancestry. By applying ADMIXTURE^23^ with *K* = 4 and then RelateAdmix^24^ to these 584 Greenlandic individuals combined with the 50 Danish individuals, we then identified and removed 384 close relatives (Figure S1A, available online) to obtain a data set without closely related individuals. Finally, by principal-component analysis (PCA) of the remaining individuals, we identified and removed nine individuals who did not cluster with the rest of the individuals from the same overall region, i.e., the north (Qaanaaq), west (Upernavik villages), south (South villages), and east (Tasiilaq villages and Tasiilaq) (Figure S1B). The latter was intended to remove putative recent migrants between the different regions of Greenland. The identified putative migrants fit well with known recent migrations in Greenland. For example, it is well known that there are recent migrants between Qaanaaq and Upernavik. In fact, 9% of participants in Kullorsuaq (the northern-most settlements in the Upernavik district) stated that they were born in Avanersuaq (the North Greenlandic county where Qaanaaq is the main settlement), and 2% of participants in Avanersuaq were born in the Upernavik district. All in all, this left us with 191 individuals from five locations in Greenland. To form the restricted Greenlandic data set combined with Danish samples, we extracted data for these 191 individuals and for the 50 Danish individuals from the full data set. Subsequently, using a step size of ten SNPs, we removed strong LD from the data set by removing SNPs such that no pair of SNPs had *r*^2^ greater than 0.5 in windows of 100 SNPs. This filtering process resulted in a data set with 31,992 SNP sites.

#### The Restricted Greenlandic Data Set Combined with HapMap Samples

Genotypes from the Greenlandic individuals included in the restricted Greenlandic data set combined with Danish individuals were merged with MetaboChip genotypes of the four original HapMap populations: CEU (Utah residents with ancestry from northern and western Europe from the CEPH collection), JPT (Japanese in Tokyo, Japan), CHB (Han Chinese in Beijing, China) and YRI (Yoruba in Ibadan, Nigeria). We removed the offspring of the HapMap trios such that all individuals in the data set were unrelated. Subsequently, all SNPs with a MAF below 5% and missingness above 1% were removed, leaving data for 102,559 SNP sites. No LD pruning was performed for this data set. We note that the *D* statistics estimated from this data set are consistent with the claim that the Greenlanders in this restricted data set have no European admixture (Figure S2).

### Sequencing Data

For estimation of site-frequency spectra (SFSs) and sequence-data-based F_ST_, we used the exome sequencing data generated by Moltke et al.^8^ from the 18 parents of nine trios of Greenlanders with no Danish ancestry from Qaanaaq (three trios), Tasiilaq villages (five trios), and Upernavik villages (one trio). Additionally, we downloaded 18 unrelated HapMap samples from four different populations (CEU, JPT, CHB, and YRI) from 1000 Genomes.^25^ For the exome data, the extended target region for Agilent SureSelect spanned 75 Mb. These regions were used for both the whole-genome sequencing data from 1000 Genomes and the exome sequencing data.

### Admixture and PCA

Admixture proportions were estimated with the ADMIXTURE software^23^ with a range of *K* values (the assumed number of ancestral populations). For each *K* value, we ran ADMIXTURE 100 times with different seeds in order to evaluate convergence. For all values of *K*, ADMIXTURE converged to the same (largest) likelihood in more than 50% of these 100 runs. The estimated ancestry proportions for different *K* values were plotted together and colored in a manner that minimized the mean root-squared error between the different plots. PCAs were performed on the basis of the model presented in Patterson et al.,^26^ and the results were colored on the basis of location.

### Estimation of Admixture-Corrected Allele Frequencies

Several of our analyses were based on Greenlandic allele frequencies corrected for European admixture. To estimate admixture-corrected allele frequencies, we first extracted the European ancestry proportions estimated by ADMIXTURE^23^ under the assumption of two ancestral populations (*K* = 2). To model the uncertainty associated with sampling individuals from a population, we parameterized the discrete distribution of admixture proportions, for each Greenlandic population, by using a mixture of a point mass at 0, a beta distribution, and a point mass at 1. We therefore had four parameters to estimate: the fraction of individuals with a European ancestry proportion of 0, the fraction of individuals with a European ancestry proportion of 1, and the two parameters of the beta distribution. We computed maximum-likelihood estimates of these parameters. Figure S3 shows the comparison between the average of the observed values and the expectation from the modeled distribution of admixture proportions. Finally, we used the discrete estimated distribution of admixture proportions for each population to compute the Greenlandic allele frequencies without the contribution of genetic admixture with Europeans. For each site, we computed the admixture-corrected Greenlandic allele frequencies *f′*_G_ as

$$f_{\text{G}}^{\prime }=\sum_{b=1}^{B}\frac{f_{\text{G}}-\alpha_{b}f_{\text{D}}}{1-\alpha_{b}}\text{Pr}\left(\alpha_{b}\right)$$

where *f*_G_ is the admixed Greenlandic allele frequency, *f*_D_ is the Danish allele frequency, and *α*_*b*_ is the European ancestry proportion at the *b*^th^ bin of the discrete distribution estimated as described above. An arbitrary number of bins (ten) was chosen for ease of calculation.

### SFS Estimation

We estimated the SFS from sequencing data from five populations (Greenlanders, CEU, CHB, JPT, and YRI) by using the full maximum-likelihood method from Nielsen et al.^27^ as implemented in ANGSD (Analysis of Next Generation Sequencing Data).^28^ Because we only had exome data from the Greenlanders, we only included data from the extended Agilent SureSelect exome target region for all five populations. Before estimating the SFSs, we discarded reads with a mapping quality below 30 and bases with a quality score below 20, which correspond to an error rate of 0.1% and 1%, respectively.

We also estimated the 2D SFS of the Greenlandic population and CHB and of the Greenlandic population and CEU. For these spectra, we only used sites from the extended target region where we had coverage for both populations.

When estimating the SFSs with ANGSD, we chose to use the SAMtools genotype likelihood model.

### LD Estimation for Ancestral Populations

LD is affected by admixture. Therefore, we developed a model that can accommodate admixture by first estimating the haplotype frequencies in each ancestral population from the observed genotypes and then calculating LD between pairs of SNPs from these haplotype frequencies.

We estimated the haplotype frequencies by using a maximum-likelihood approach: let $G=\left(G_{1},G_{2},\;...,G_{n}\right)$ be the genotypes of *n* individuals and $G_{i}=\left(G_{i}^{1},G_{i}^{2}\right)$ be the genotypes of individual *i* at the pair of SNP sites of interest. Further, assume that all *n* individuals have ancestry from one or more of *K* ancestral populations and that we know the admixture proportions $\alpha_{i}=\left(\alpha_{i}^{1},\alpha_{i}^{2},\ldots ,\alpha_{i}^{K}\right)$ for each individual *i*, and let α denote the vector $\left(\alpha_{1},\alpha_{2},\ldots ,\alpha_{n}\right)$ (see previous section for a description of how we inferred the admixture proportions). Finally, let the frequency of haplotype *j* for the *k*^th^ population be denoted as $p_{j}^{k}$, and let *h =* (*h*_1_, *h*_2_) be the unobserved pair of haplotypes for an individual, where the two haplotypes originate from the unobserved ancestral populations *k*_1_ and *k*_2_. Then, the likelihood of the ancestry-specific haplotype frequencies $\;p=\left(p_{j}^{k}\right)$ given the observed genotypes and ancestry proportions can be written as

$$\begin{array}{c} \text{L}\left(p\right)=\mathrm{Pr}\left(G|p,\alpha \right)\\ =\prod_{i=1}^{n}\mathrm{Pr}\left(G_{i}|p,\alpha_{i}\right)\\ =\prod_{i=1}^{n}\sum_{h\in H}\sum_{k_{1}=1}^{K}\sum_{k_{2}=1}^{K}\mathrm{Pr}\left(G_{i},h_{1},h_{2},k_{1},k_{2}|p,\alpha_{i}\right)\\ =\prod_{i=1}^{n}\sum_{h\in H}\sum_{k_{1}=1}^{K}\sum_{k_{2}=1}^{K}\mathrm{Pr}\left(G_{i}|p,\alpha_{i},h_{1},h_{2}\right)\mathrm{Pr}\left(h_{1},h_{2}|p,k_{1},k_{2}\right)\mathrm{Pr}\left(k_{1},k_{2}|\alpha_{i}\right)\\ =\prod_{i=1}^{n}\sum_{h\in H}\sum_{k_{1}=1}^{K}\sum_{k_{2}=1}^{K}\mathrm{Pr}\left(G_{i}|h_{1},h_{2}\right)p_{h_{1}}^{k_{1}}p_{h_{2}}^{k_{2}}\alpha_{i}^{k_{1}}\alpha_{i}^{k_{2}}\\ =\prod_{i=1}^{n}\sum_{h\in h\left(G_{i}\right)}\sum_{k_{1}=1}^{K}\sum_{k_{2}=1}^{K}p_{h_{1}}^{k_{1}}p_{h_{2}}^{k_{2}}\alpha_{i}^{k_{1}}\alpha_{i}^{k_{2}} \end{array}$$

In the above, we assume that the ancestral population of a haplotype is the same at the two SNP sites, given that these sites are in close proximity along the genome. Furthermore, we denote the set of all possible pairs of haplotypes by *H*, whereas we denote the set of all pairs of haplotypes that are consistent with the genotypes of individual *i* by *h*(*G*_*i*_). The last rewriting step follows from the observation that Pr(*G*_*i*_|*h*_*1*_*,h*_*2*_) is equal to 1 if the genotypes *G*_*i*_ are consistent with the haplotypes *h*_*1*_ and *h*_*2*_ (i.e., *h* belongs to *h*(*G*_*i*_)) and that Pr(*G*_*i*_|*h*_*1*_*,h*_*2*_) is equal to 0 otherwise.

We obtain maximum-likelihood estimates of the ancestry-specific haplotype frequencies by maximizing the above likelihood. This is done efficiently with the following expectation-maximization (EM) algorithm. First, random starting points are sampled from a uniform distribution. Then, EM iterations are performed until each new step does not change the parameters (we used a tolerance of 10^−6^), and each EM iteration for each haplotype frequency is given as

$$p_{j*}^{k}=\frac{1}{2n}\sum_{i=1}^{n}\frac{\sum_{h\in H\left(G_{i}\right)}\sum_{k_{1}=1}^{K}\sum_{k_{2}=1}^{K}p_{h_{1}}^{k_{1}}p_{h_{2}}^{k_{2}}\alpha_{i}^{k_{1}}\alpha_{i}^{k_{2}}\left(I_{j}\left(h_{1}\right)I_{k}\left(k_{1}\right)+I_{j}\left(h_{2}\right)I_{k}\left(k_{2}\right)\right)}{\sum_{h\in H\left(G_{i}\right)}\sum_{k_{1}=1}^{K}\sum_{k_{2}=1}^{K}p_{h_{1}}^{k_{1}}p_{h_{2}}^{k_{2}}\alpha_{i}^{k_{1}}\alpha_{i}^{k_{2}}\left(I_{k}\left(k_{1}\right)+I_{k}\left(k_{2}\right)\right)},$$

where *I* is the indicator function.

### Inbreeding Estimation

Admixture also affects standard estimators of inbreeding coefficients, and we correct for this by allowing for admixture. We estimate the inbreeding coefficient *F* for each individual with a maximum-likelihood method that uses the estimated admixture proportions $\alpha =\left(\alpha^{1},\alpha^{2},\ldots ,\alpha^{K}\right)$ for the given individual along with the allele frequencies for the *K* source populations $f_{s\;}=\left(f_{s}^{1},f_{s}^{2},\ldots ,f_{s}^{K}\right)$ for each site *s* as estimated by ADMIXTURE.^23^ Let $g_{s}\in \left(0,1,2\right)$ be the individual’s genotype at site *s*, and let $f_{s}^{*}=\;\sum_{k=1}^{K}\alpha^{k}f_{s}^{k}$ be the probability of observing the minor allele at site *s*. The likelihood of *F* given the genotype data, $D=\left(g_{1},g_{2},\ldots ,g_{S}\right)$, is then given as

$$\text{L}\left(F\right)=\text{Pr}\left(D|F\right)=\;\prod_{s=1}^{S}\text{Pr}\left(g_{s}|F\right)$$

with

$$\text{Pr}\left(g_{s}\text{|}F\right)=\;\{\begin{array}{c} \;{\left(1-f_{s}^{*}\right)}^{2}\left(1-F\right)+\left(1-f_{s}^{*}\right)F,\;\;g_{s}=0\\ \;2\left(1-f_{s}^{*}\right)f_{s}^{*}\left(1-F\right),\;g_{s}=1\\ \;f_{s}^{*}f_{s}^{*}\left(1-F\right)+f_{s}^{*}F,\;\;g_{s}=2 \end{array}$$

The maximum-likelihood estimate of *F* is then the *F* value that maximizes the above likelihood.

### TreeMix Analyses

We performed TreeMix^29^ analyses of allele frequencies estimated from two different data sets: the full data set without LD and the restricted Greenlandic data set combined with HapMap samples.

For the TreeMix analysis of the allele frequencies estimated from the full data set without LD, the allele frequencies were corrected for European ancestry before the analysis was performed. Then, 100 trees were generated with different seeds. Except for a few, they all had the same (highest) likelihood. In the cases where all trees did not have the same likelihood, the tree with the highest likelihood was used. Because this data set was pruned for LD, a window size of one SNP was used.

Ten trees were generated for the TreeMix analysis of the allele frequencies estimated from the restricted Greenlandic data set combined with HapMap samples. All gave the same likelihood. A window size of 100 SNPs was used for accommodating LD; however, increasing the window size to 500 or decreasing to 50 did not change the topology.

### *D* Statistics

We performed *D*-statistic-based tests on the SNP chip data from the restricted Greenlandic data set combined with HapMap samples. First, we estimated the allele frequency in each genotyped site separately for the population in each location. Then, we estimated the *D* statistics as

$$D\left(\text{H}1,\text{H}2;\text{H}3,\text{H}4\right)=\frac{\sum_{i=1}^{M}\left(f_{i}^{\text{H}3}-f_{i}^{\text{H}4}\right)\left(f_{i}^{\text{H}1}-f_{i}^{\text{H}2}\right)}{\sum_{i=1}^{M}\left(f_{i}^{\text{H}3}+f_{i}^{\text{H}4}-2f_{i}^{\text{H}3}f_{i}^{\text{H}4}\right)\left(f_{i}^{\text{H}1}+f_{i}^{\text{H}2}-2f_{i}^{\text{H}1}f_{i}^{\text{H}2}\right)},$$

where H1, H2, H3, and H4 represent populations in the tree (((H1, H2), H3), H4), where H4 is the outgroup, *M* is the number of sites included, and $f_{i}^{\text{H}1}$ is the allele frequency for population H1 at site *i*.^30^ Only sites with information for all four populations were included. *Z* scores were obtained from the *D* statistics with SEs based on a “delete m jackknife for unequal m” procedure^31^ for 5 Mb regions weighted according to the number of SNPs in each block.

### Inference of Ancestry Tract Lengths

Genotypes from the full data set were phased with shapeit2^32^ with the 1000 Genomes phased variant panel (Phase I v.3) as the reference panel. HapMap recombination rates (Phase II b37) were used as a proxy for the human genome genetic map. Local ancestry was inferred with RFMix^33^ for Qaanaaq and South Village individuals with an estimated global European ancestry proportion greater than 0.05 according to the ADMIXTURE analysis described above. We used two ancestral reference populations. As a proxy for the Inuit ancestral population, we used a reference panel (n = 46) composed of Greenlandic individuals with a global European ancestry proportion less than 0.05 (mean European ancestry = 0.0038; maximum European ancestry = 0.0436). We used the Danish samples (n = 46) as a reference panel to represent the European ancestral population. Local ancestry was inferred for all admixed Qaanaaq and South village individuals jointly with the RFMix admixture timing parameter G = 20, which corresponds to admixture occurring at least 500 years ago and a generation time of 25 years, and a window size of 0.1 cM. We allowed phase correction and used three iterations of the EM algorithm with reference panels included. To control for differences in population-level admixture proportions between Qaanaaq and South villages, we matched individuals according to ancestry proportion as closely as possible, resulting in a matched set of 40 individuals with a European ancestry proportion of at least 0.05 from each population. Length distributions of European admixture tracts were calculated for this matched set, summarized in 5 cM bins, and compared between the two populations.

The probability of observing at least one tract of length X cM in an individual, as a function of the time since admixture, can easily be approximated with the Markov approximation to tract lengths introduced by Pool and Nielsen.^40^ The tract lengths can be described by a two-state Markov process with transition rates λ_1_ = (1 − *m*)*r(t* − 1) and λ_2_ = *mr*(*t* − 1), *t* > 1 from admixed to unadmixed DNA and from unadmixed to admixed DNA, respectively. Here, *m* is the admixture proportion, *r* is the recombination rate per base pair, and *t* is the admixture time in number of generations. The probability that site *j* initiates a run of at least *k* admixed sites is *R* = π_2_λ_2_(1 − λ_1_)^k−1^, where π_2_ is the stationary probability of the unadmixed state (1 − *m*). If *R* is small and the length of the genome, *S*, is large, the probability of observing no runs of length *k* is then approximately *e*^−*RS*^ = exp[*S*(*m* − 1)*mr*(1 + (*m* − 1)*r*(*t* − 1))^(*k*−1)^(*t* − 1)]. With a genome size of *S* = 2.7 Gb and a recombination rate of 1.3 × 10^−8^ per base pair, the probability of observing no fragments larger than 39 cM is then equal to 0.9945 for an admixture fraction of *m* = 0.05 and an admixture time of *t* = 25. Equivalently, the probability of seeing at least one fragment of length 39 cM is ∼0.005 if the admixture time is 25 generations.

### Ethical Considerations

The Greenlandic samples used in this study were donated by Greenlandic individuals as part of the general public health surveys presented in Jørgensen et al.,^18^ Bjerregaard et al.,^19^ and Bjerregaard.^34^ Ethics approval for genotyping the samples and using the genotype data for public health studies was received from the Commission for Scientific Research in Greenland as a part of the study by Moltke et al.^8^ The use of the genotype data for the present study has also received ethics approval from the Commission for Scientific Research in Greenland (project 2014-08, reference 2014-098017).

## Results

To investigate the genetic history of the present-day Greenlandic population, we analyzed genetic data from 4,127 Greenlandic individuals from 15 different locations in Greenland (Figure 1), 547 Greenlandic individuals living in Denmark, 50 Danish individuals, and 208 unrelated individuals from the original HapMap project. All of these individuals were genotyped for 196,224 SNPs on the Illumina MetaboChip, and a small subset of them were exome sequenced as well. The Danish individuals were included to represent the European ancestors of the Greenlanders, which are mainly from Denmark and Norway, and the HapMap individuals were included for comparison to other populations from the rest of the world. Note that some of the results presented below are based on analyses of SNP chip data from all 4,127 Greenlanders and the 50 Danes. Other results are based on analyses of SNP chip data from a restricted subset of the Greenlanders. This subset consists of individuals who are not closely related, do not have any European ancestry (<5% estimated European ancestry), and have not recently migrated within Greenland. Because most of the 15 locations had very few such individuals, only individuals from Qaanaaq (north), Upernavik villages (west), South villages (south), Tasiilaq (east), and Tasiilaq villages (east) were included in these latter analyses. In the following sections, we will refer to the two data sets on which the below results are based as (1) the full data set and (2) the restricted Greenlandic data set. For details about the data sets, including how many of the 196,224 SNP sites did not pass filtering prior to the different analysis, see the Material and Methods.

### Recent European Gene Flow and Population Structure

**Figure 3:**
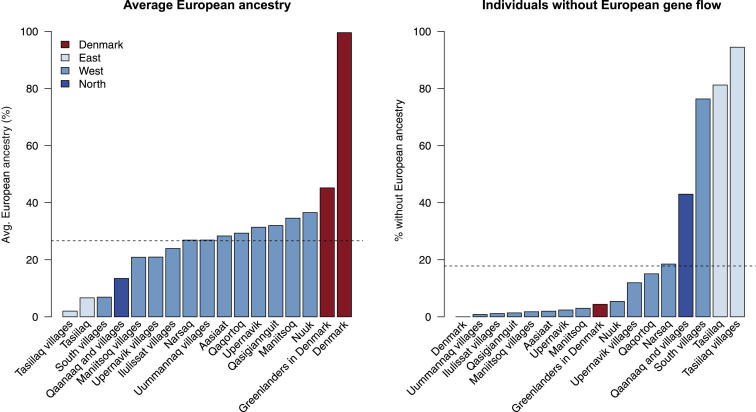
Extent of European Ancestry at Different Locations in Greenland The results shown are summaries of the admixture proportions estimated for all individuals included in this study (the full data set) under the assumption of two ancestral populations (*K* = 2). The bars in the left plot show the average European ancestry proportion at each sampling location, and the dashed line shows the average for the entire data set. The bars in the right plot show the fraction of individuals without European admixture for each sampling location, and the dashed line shows the fraction for the entire data set. The color scheme is the same as in Figure 1.

Using a subset of the genotyped Greenlandic individuals (2,733 individuals from the IHIT cohort), we previously showed in a study focused on disease mapping that there has been a large amount of gene flow from Europe into Greenland and that most Greenlanders have both European and Inuit ancestry.^8^ To further explore the genetic structure of the Greenlandic population, we here estimated admixture proportions for the full data set by using the program ADMIXTURE^23^ and stratified the results according to location. First, we assumed that the Greenlandic individuals have ancestry from two ancestral populations (*K* = 2), so all Danish individuals were assigned one ancestral population, and the Greenlandic individuals were assigned a mixture of both ancestral populations (Figure 2). We interpreted the two ancestry components of the Greenlandic individuals to be European ancestry and Inuit ancestry. In doing so, we found that there has been gene flow from Europeans into most locations in Greenland and that more than 80% of Greenlanders have European ancestry (Figures 2 and 3). On average, the Greenlanders have ∼25% European ancestry; however, some locations in Greenland have a considerably smaller amount of European ancestry. Specifically, participants from Tasiilaq in East Greenland, the small villages in South Greenland (South villages), and Qaanaaq in North Greenland (Thule) have less European ancestry. In fact, most individuals in Tasiilaq and the South villages have only Inuit ancestry (Figure 3).

To investigate the population structure within the Inuit ancestry, we inferred ancestry proportions with higher numbers of assumed ancestral populations (*K* = 3–5). When three ancestral populations (*K* = 3) were assumed, the Danes were again assigned one ancestry component, but Greenlanders in Qaanaaq in North Greenland and in Tasiilaq in East Greenland were also each assigned their own component (Figure 2). The rest of the Greenlandic locations were inferred to be mixtures of all three components. When four ancestral populations (*K* = 4) were assumed, the results remained similar, except in this case, all the Greenlandic locations other than Qaanaaq and Tasiilaq were inferred to be mixtures of all four components. These results do not support the claim of a shared genetic component between North and East Greenlanders,^13^ but it fits well with the geographic regions in Greenland, where the two geographically extreme locations are Qaanaaq in the north and Tasiilaq in the east. Both of these locations are fairly isolated from the west and south of Greenland, where most Greenlanders live. The physical distance between Tasiilaq and Qaanaaq and the rest of the locations might also explain why these locations have less gene flow from Europe. Further increasing the number of assumed ancestry components (*K* = 5), we found that the areas around Upernavik and Maniitsoq also received their own predominant ancestry component. Interestingly, the South village population was not assigned a unique ancestry component but was the only West Greenlandic population to be assigned a large amount of Tasiilaq ancestry. For the analyses performed with higher *K* values (*K* > 2), it should be noted that care should be taken when results are interpreted because the model underlying the program ADMIXTURE might not represent the nature of the data well. First, the fact that none of the individuals from Upernavik villages and Maniitsoq villages were inferred to be 100% from the components that are predominant in these locations at *K* = 5 could be an indication that this *K* value is too high. Second, the fact that the individuals from West Greenland were inferred to have ancestry from both Qaanaaq and Tasiilaq under the *K* = 3–4 models does not necessarily indicate that the West Greenlanders are admixed. These results could also be caused by a scenario where Greenland was settled by Inuit who entered North Greenland and from there migrated to South Greenland along the west coast and from there to East Greenland. We will return to this point later.

**Figure 4:**
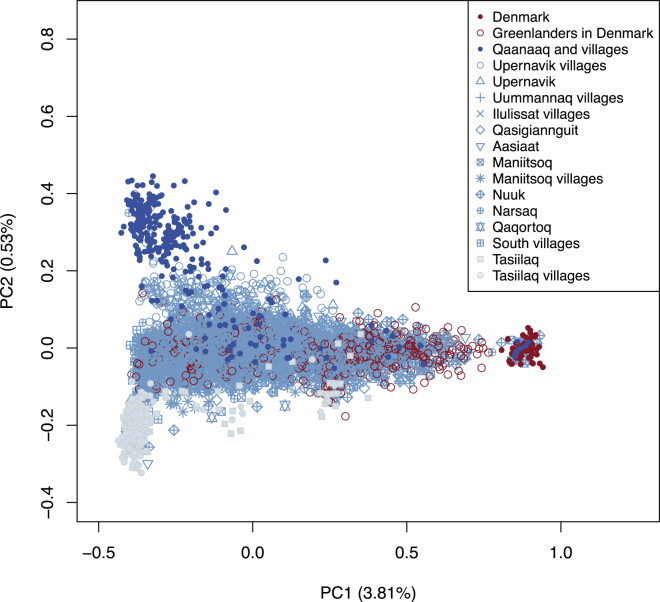
PCA of the Full Data Set The first two principal components (PC1 and PC2) based on a PCA of the genetic covariance matrix of all the Greenlandic and Danish individuals included in the study. The estimated percentages of the variation explained by the two principal components are shown in the axis labels. The color scheme is the same as in Figure 1.

We also visualized the population structure by using PCA. As can be seen in Figure 4, there are three extreme locations: Denmark representing Europe, Tasiilaq representing East Greenland, and Qaanaaq representing North Greenland. The first principal component reflects an Inuit-to-Europe gradient, whereas the second principal component reflects a within-Inuit gradient from north to east and with intermediate populations in the south. The existence of such a gradient could suggest that modern East Greenlanders are descendants of people who first migrated from north to south along the west coast of Greenland. An alternative explanation is that the South Greenlanders are admixed between East and West Greenlanders and that East Greenlanders are descendants of a separate wave of migration from the north down the east coast. We will return to this point in the section on migration routes.

To further investigate the population structure within the Inuit ancestry, we also inferred ancestry proportions and performed PCA of the restricted Greenlandic data set combined with Danish samples. The inferred ancestry proportions are shown in Figure S4, in which the structure among Inuit is clearly visible. However, it is not sufficiently pronounced for each location to be assigned a unique ancestry component. Even though Upernavik villages and South villages represent the extreme ends of West Greenland (including South Greenland), they were not assigned two different components. Instead, they were assigned the same component, although individuals from Upernavik villages harbor a substantial fraction of the Qaanaaq ancestry component. The PCA in Figure S5 suggests the same: Upernavik villages and South villages cluster closely together even though they are physically located far from each other. These results are consistent with F_ST_ values estimated by the Weir and Cockerham estimator^35^ from the same restricted Greenlandic data set combined with HapMap samples (Table S1): F_ST_ is a measure of how different populations are genetically, and the fact that the estimated F_ST_ between Upernavik villages and South villages is smaller than the estimated F_ST_ between Upernavik villages and any of the two other locations suggests that Upernavik villages are genetically closer to South villages than to the other locations.

### Sex-Biased Gene Flow

Bosch et al.^6^ analyzed mtDNA and Y chromosome DNA from 69 Inuit and demonstrated that the mtDNA, which is maternally inherited, was exclusively of Inuit origin, whereas more than 50% of the Y chromosomes, which are paternally inherited, were of European origin.^6^ However, their study was based on a small number of individuals from a limited number of locations in Greenland. To more broadly assess and quantify the sex bias in the European gene flow, we estimated the amount of mtDNA of European origin in our much larger full data set. We distinguished between European and Inuit mtDNA by using a single diagnostic mtDNA SNP: the MT1736 marker that defines the A haplogroup. This marker perfectly separates the two populations for all unadmixed individuals (Table S2), and we used it to obtain estimates of the amount of mtDNA of European origin shown in Figure S6. We found that although the mtDNA in Greenland is not exclusively Inuit, the amount of European female ancestry based on mtDNA is only ∼1.0%. This is about 25 times lower than the proportion of autosomal DNA of European origin. The large discrepancy between admixture proportions at autosomal and mtDNA markers is in line with the results of Bosch et al.,^6^ who concluded that 50% of the male ancestry in Greenland is European.

### Consequences of Being a Small and Historically Isolated Population

The Inuit have not experienced the same population growth as many of the standard reference populations, such as Han Chinese and Europeans. Furthermore, they might have lived in relatively small subpopulations and undergone a series of bottlenecks as they colonized the Arctic. For this reason, we would expect the Greenlanders to have a relatively small effective population size in comparison to East Asian or European populations.

**Figure 5:**
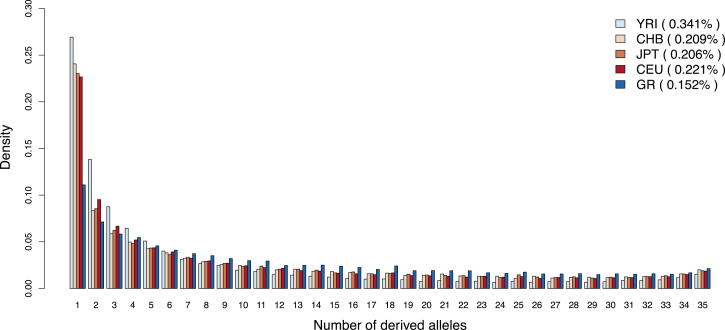
SFSs for Greenlanders and Four HapMap Populations The SFSs for 18 Greenlanders (36 chromosomes) without European ancestry, here denoted GR, are compared with the SFSs for 18 individuals from each of the four original HapMap populations: CEU (European ancestry), JPT (Japanese ancestry), CHB (Han Chinese ancestry), and YRI (African ancestry). All SFSs were estimated from sequencing data. The Greenlanders were exome sequenced, and the HapMap populations were whole-genome sequenced as part of the 1000 Genomes Project. Only the 75 Mb extended target regions defined by Agilent SureSelect were used for all five populations. The estimated variability (fraction of polymorphic sites) in each of the populations is shown in parentheses in the legend.

Populations with historically small effective sizes are expected to harbor less nucleotide variability than larger populations. To assess whether this is the case for the Greenlandic population, we estimated the SFS for the Greenlandic population by using exome sequencing data generated by Moltke et al.^8^ from 18 parents from nine trios of Greenlanders with no Danish ancestry. We also estimated SFSs for four HapMap^22^ populations (CEU, JPT, CHB, and YRI) by using data from 18 unrelated individuals from each of these populations, which were sequenced as part of the 1000 Genomes Project.^25^ From these five SFSs, we then estimated variability levels for each of the five populations (Table S3). Most notably, this table shows that the variability, measured as the fraction of polymorphic sites, is markedly lower in Greenland than in the other populations. Additionally, the SFSs show that the Greenlandic population harbors proportionally fewer rare variants than the four HapMap populations (Figure 5). Both observations are consistent with a history of small population sizes, isolation, and founder events. We note that the reason we used (exome) sequencing data instead of SNP chip data for the above comparison is that the MetaboChip is biased toward SNPs with high frequency in European populations, and this ascertainment bias could strongly affect the results of a comparison between Greenlanders and other populations, especially Europeans. However, because the SNP ascertainment bias should affect all Greenlandic locations equally, the SNP chip data can be used for comparing nucleotide variation levels between the different locations in Greenland. We did this by estimating nucleotide variation levels, measured as mean MAF, for each Greenlandic location from the full data set without LD (Figure S7). To account for the European admixture, we corrected the allele frequencies for the estimated European ancestry (see Material and Methods for details). Interestingly, a slight decay of genetic variation following a gradient from north to west to south to east can be observed among the Greenlandic locations (Figure S8), which could again indicate support for only one migration wave that moved from north to west to south to east.

**Figure 6:**
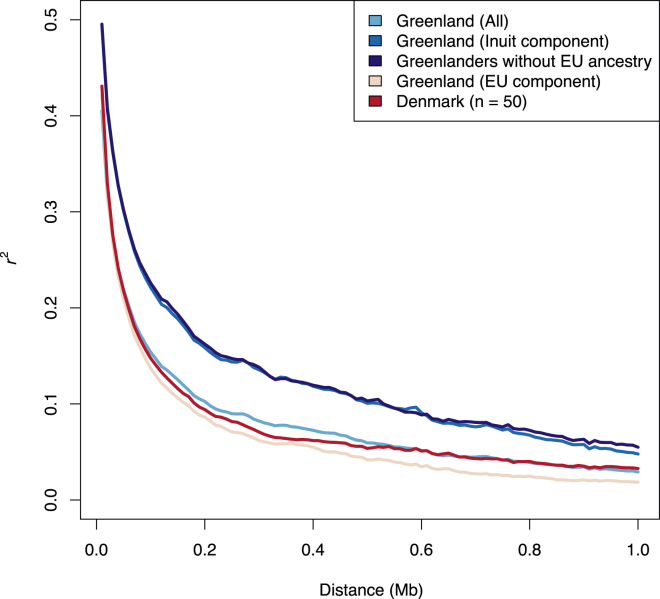
LD Decay for Different Populations The average LD (*r*^2^) is shown as a function of the physical distance on autosomes. LD was estimated in Danes, Greenlanders without European admixture, and all Greenlanders (the full data set was used, and admixture was ignored). Additionally, LD in all Greenlanders (the full data set) was estimated with a method that takes admixture into account. This was done under the assumption of two ancestral populations, and the estimates correspond to estimates for the ancestral European population (EU component) and the ancestral Inuit population (Inuit component). Note that only 50 Danes were used for the estimate of LD for Danes, which is why the mean *r*^2^ is higher at greater distances for the Danes than for the European component of the Greenlandic population. For each subset of the data, a minor allele cutoff of 5% was used.

To investigate whether historical demography has had an effect on LD in the Greenlandic population, we estimated LD in the Greenlandic individuals and compared it to LD patterns in the Danes, both on the basis of data from the full data set (Figure 6). LD among the Greenlanders was markedly higher than among the Danes, which has also been indicated by previous studies.^9^ However, because of the recent European admixture, these LD estimates do not only reflect the more ancient demographic history of the Greenlandic population. To correct the LD estimates for the admixture and provide LD estimates for the ancestral Inuit and European populations, we also inferred the ancestral haplotype frequencies from the Greenlandic individuals. The model used for inferring the ancestral haplotype frequency assumes that the ancestry is the same for both alleles on the same haplotype and that the ancestries of an individual’s haplotype are conditionally independent on the admixture proportions. The consequence of violating these assumptions seems to have a minimal impact given that the estimates from the unadmixed individuals are similar to their inferred ancestral haplotype frequency (Figure 6). The analyses showed that LD in the ancestral Inuit population was markedly higher than the LD in the present-day Greenlandic population, whereas the ancestral European population had approximately the same amount of LD as the 50 present-day Danish individuals from the full data set (Figure 6). Thus, the recent European admixture has reduced the LD of the Greenlanders significantly. The decreased LD due to gene flow from Europe might seem somewhat counterintuitive, given that admixture creates LD where there previously was none. However, when a population with high LD mixes with a population with lower LD, the resulting population can have intermediate or even lower levels of LD. For example, if two SNPs are in perfect LD in one population, then gene flow from another population without perfect LD will always result in a decrease in LD. This scenario can clearly be seen in Figure S9, where perfect haplotype blocks are present in the Inuit component but almost absent in the European component.

Finally, in addition to having a historically small population size, Inuit populations have traditionally lived in small groups, where the probability of mating with a comparatively closely related partner is increased. To investigate to what extent this has affected the population genetically, we estimated inbreeding coefficients for all the individuals and stratified the results according to location (Figure S10). If no admixture correction was performed, the inbreeding coefficients in some locations were estimated to be extremely high with an average value above 0.13. However, after correction for admixture, the average inbreeding coefficients were similar among locations in Greenland; they ranged from *F* = 0.008 to *F* = 0.014 and were comparable to coefficients estimated for the Danes (*F* = 0.007). The individuals with the lowest amount of inbreeding were Greenlanders living in Denmark.

The above results suggest that the Greenlandic population is indeed affected by being a historically small and isolated founder population in several ways. However, we note that the population stands out in at least one important way in comparison to well-studied founder populations, such as the Finnish and the Icelandic populations: these other founder populations are all genetically similar to at least one large population, whereas the Inuit are not closely related to any large population. For example, estimates of genetic differentiation are very low between the Icelandic population and both the Norwegian population (F_ST_ = 0.0016) and the Scottish population (F_ST_ = 0.0020).^36^ For comparison, on the basis of our SNP chip data, we estimated F_ST_ to be 0.12 between the Greenlandic population and the Han Chinese (CHB) HapMap samples (Table S1), and the F_ST_ estimate based on sequencing data for the same two populations, which do not suffer from SNP ascertainment bias, was also 0.12 (Table S3). F_ST_ between locations in Greenland and Europe ranged from 0.15 to 0.17 for the SNP chip data (Table S1) and was estimated to be 0.16 for the sequencing data (Table S3). We note that these values are higher than the recently reported F_ST_ values ranging from 0.039 to 0.101.^9^ The large difference in F_ST_ values between locations in Greenland reported by Pereira et al.^9^ and the difference between their estimates and ours are most likely a result of the fact that Pereira et al. did not exclude European admixture when estimating F_ST_, whereas our estimates are based only on data from Greenlandic individuals without any European ancestry. This observed difference between the Greenlandic population and other founder populations, such as the Icelandic and Finnish, is most likely due to the fact that the Greenlanders’ ancestral Inuit population was an isolated and small population for a longer period of time than these other populations.

### Coastal Migration Route

The inferred admixture proportions and the estimated geographic variation in levels of nucleotide variation both suggest a model of Greenland settlement from the north to the south and subsequently from the south to the east, given that there is no evidence of shared genetic components between the east (Tasiilaq) and the north (Qaanaaq) or between the east and the northwest (Upernavik). SNP-chip-based F_ST_ estimates from individuals without European ancestry (based on the restricted Greenlandic data set combined with HapMap samples) are also consistent with this model: Tasiilaq in East Greenland is genetically furthest away from Qaanaaq in North Greenland and closest to villages in South Greenland (F_ST_ = 0.04 and 0.02, respectively, see Table S1).

**Figure 7:**
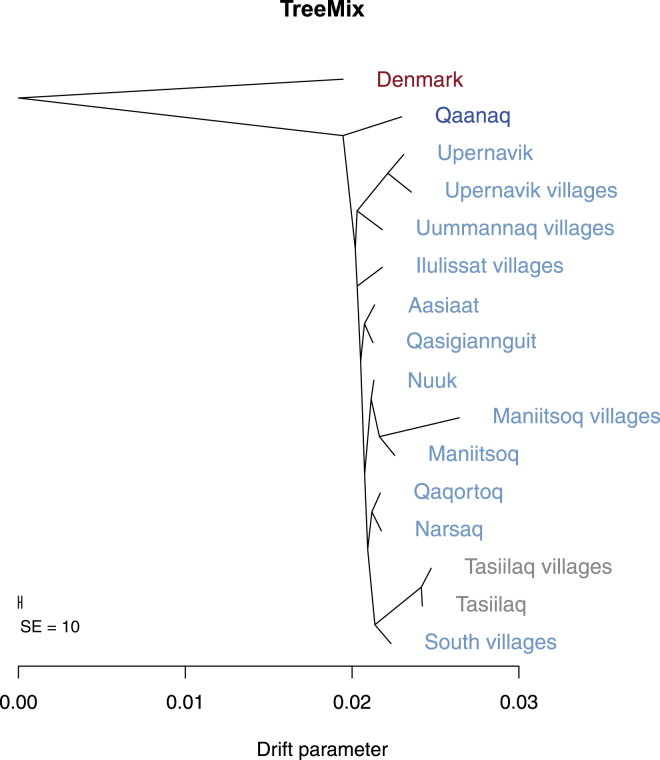
Maximum-Likelihood Tree Relating Individuals from All the Different Sampling Locations The tree was estimated by TreeMix from allele frequencies that were estimated from the full data set without LD and corrected for European admixture. The color scheme is the same as in Figure 1, except that the lightest blue color has been replaced by gray so that it is easier to read.

To investigate this further, we used TreeMix^29^ with Danish individuals as an outgroup to root the tree to infer the maximum-likelihood genetic-drift tree topology relating people from the different locations (Figure 7). We performed this analysis by using allele frequencies estimated from the full data set without LD and corrected for European admixture. The resulting tree is consistent with a single coastal route migration in which each location sequentially splits off along the coastline from the north to the south and subsequently from the south to the east. We obtained a similar TreeMix tree when we performed the same analysis with the restricted Greenlandic data set combined with HapMap samples (this data set includes only Greenlandic individuals with no European ancestry, no close relatives, and no recent migrations between Greenlandic regions; Figure S11). The topology of this tree differs in one respect, though: the placement of the root of the Greenlandic subtree. Whereas the tree inferred from the full data set without LD has Qaanaaq as an outgroup, this tree has the root placed so Upernavik villages are on the same side of the root as Qaanaaq. However, the amount of drift from the root to the split between Qaanaaq and Upernavik villages is at the same time inferred to be very small, which means that this TreeMix result is also consistent with a single migration event. Note that we also tried to run TreeMix on both data sets while allowing for one admixture event. However, the results were inconclusive and seemed likely to reflect artifacts of the method rather than real admixture events, and we therefore have not included them here.

**Figure 8:**
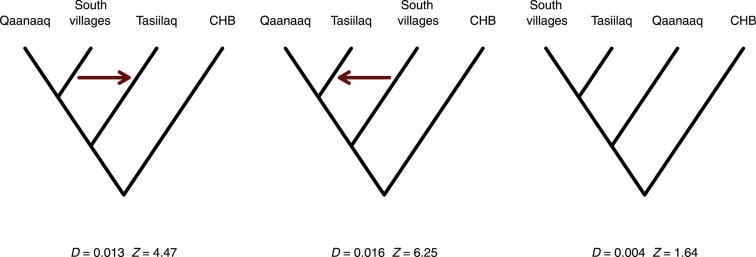
*D* Statistics for Different Possible Topologies CHB are the Han Chinese HapMap individuals, *D* is the statistic, and *Z* is a standard score (*Z* score), which is usually considered significant with an absolute value above 3. The first two topologies were rejected, whereas the last was not. Using Upernavik villages instead of South villages also gave a nonsignificant result (*Z* = −0.14). Thus, we cannot reject that Qaaqaaq is an outgroup, consistent with the single-wave coastal route. Suggested gene flows that could explain the rejection of the topology are shown as red arrows. The results shown are based on the restricted Greenlandic data set combined with HapMap samples (this data set includes Greenlandic individuals who are not closely related, do not have any recent European ancestry, and have not recently migrated within Greenland).

To formally test the single coastal migration event, we used *D* statistics^30^ estimated from the restricted Greenlandic data set combined with HapMap samples. As shown in Figure 8, this led to the rejection of all tree topologies in which Qaanaaq was not an outgroup to the South villages and Tasiilaq. The same results were obtained when Qaanaaq was replaced by Upernavik villages (Figure S12). If Tasiilaq (East Greenland) was reached via a migration route along the northern coast of Greenland, and thus from the north rather than the south, we would not expect Tasiilaq and the South villages to form an ingroup to both Qaanaaq and Upernavik villages. Likewise, if there were migrations to the east from both the south and the north, then we would expect to reject the topologies where Qaanaaq or Upernavik villages were the outgroup. Hence, the *D*-statistic-based test results provide further support for the single coastal migration route.

### Admixture with the Dorset and the Norse Vikings in Greenland

The results from the *D*-statistic-based tests mentioned above also suggest that the Inuit did not, as hypothesized by Helgason et al.,^13^ interbreed with the Dorset in East Greenland. If individuals in Tasiilaq had ancestry from a previous migration, e.g., the Dorset, then we would expect all trees where Tasiilaq is an ingroup to be rejected. However, the tree with South villages and Tasiilaq as ingroups and Qaanaaq as an outgroup (Figure 8) was not rejected. To further address this question, we also performed a more direct *D*-statistic-based test of admixture. It has recently been shown with ancient DNA that individuals from the Saqqaq and the Dorset cultures are genetically similar.^10^ Therefore, using the high-coverage genome of a ∼4,000-year-old sample from the Saqqaq culture,^7^ we can test for Dorset admixture in East Greenland by estimating *D* statistics for topologies with a Greenlandic location in East Greenland (Tasiilaq or Tasiilaq villages) and a Greenlandic location in the rest of Greenland (Qaanaaq and South villages) as ingroups and the Saqqaq sample as an outgroup. If the Inuit in East Greenland interbred with the Dorset, we would expect these *D* statistics (and the *Z* values estimated from them) to differ significantly from 0. We did not find the Saqqaq sample to be significantly (*Z* > 3) closer to Tasiilaq than to Qaanaaq or South villages (Figure S13). However, one test (the test of the topology ((H1 = South villages,H2 = Tasiilaq villages),H3 = Saqqaq),H4 = CHB)) was suggestive with *D* = 0.008 and Z = 2.58, the latter of which is considered significant in some studies. On the basis of these analyses, we cannot exclude that interbreeding took place, given that the *D*-statistic-based tests used do not have full power to detect admixture events if they involve only low amounts of gene flow. However, it does suggest that the Dorset have not contributed much gene flow to the modern East Greenlanders.

The question of whether the Inuit interbred with the Norse Vikings is more difficult to answer given that the Norse Vikings were Europeans just like the later colonizers, whom we know interbred with the Inuit. Hence, to answer this question, one has to separate the recent admixture (taking place from 1721) from any potential older European admixture, which can be difficult.

One approach to address this question is to take advantage of the fact that most individuals in the south show no recent European gene flow. The largest Viking settlement was located in Southwest Greenland, and the ancestors of the individuals in the South villages passed this Viking settlement before settling in the south. Thus, if the Inuit and the Norse Vikings interbred in the west before the Inuit settled in the south or they interbred later in South Greenland, then we would expect individuals in the south to have some Norse Viking ancestry. On the contrary, it is very unlikely that the individuals in Qaanaaq would have any such ancestry given that they descend from Inuit who entered Greenland after the Norse Vikings left Greenland. If the Inuit interbred with the Norse Vikings, we would therefore expect to see signatures of ∼600-year-old European admixture in the Greenlanders in the South villages, but not in the Greenlanders in Qaanaaq. However, individuals in the South villages overall have less European ancestry than most other locations, including Qaanaaq (Figure 3), and importantly, more than half of the individuals from the South villages are estimated to have no European ancestry. Out of the 169 individuals from the South villages, only 40 are estimated to have more than 5% European ancestry. As the variance in admixture proportions among individuals decreases fast with time since admixture,^37^, ^38^ finding such a large proportion of individuals without admixture is unlikely if the time of admixture is old.

Genomes with both Inuit and European ancestry can be divided into alternating “ancestry tracts” along the length of each chromosome, and the distribution of tract lengths in an admixed population carries information about the timing of admixture and the admixture proportion in a population.^39^, ^40^, ^41^, ^42^ More recent admixture results in longer admixture tracts. To investigate whether European ancestry in the individuals who are estimated to have more than 5% European ancestry can be attributed to Norse Viking admixture, we inferred the length of European ancestry tracts in admixed Greenlander genomes. This analysis showed that admixed individuals from the South villages all have at least one European ancestry tract that is longer than 39 cM. The presence of such large European admixture tracts suggests that a substantial proportion of European admixture originated from interbreeding during the time of Danish colonization, because, as shown in the Material and Methods, the chance that an individual will harbor such a long tract if the admixture time is 25 generations is ∼0.005. However, it does not exclude the possible presence of admixture tracts originating from interbreeding with Norse Viking populations. Because inferring a short ancestry tract with certainty is very difficult, especially with data from the sparse MetaboChip, we did not directly look for specific instances of short tracts expected from more ancient admixture. Instead, we compared the tract-length distributions from Qaanaaq and the South villages. If Norse Vikings are among the ancestors of the Greenlanders in the South villages and not of the Greenlanders in Qaanaaq, we would expect to see a difference in their tract-length distributions such that the South villages have more short tracts. However, when we matched the inferred global admixture proportions between the two locations, the two tract-length distributions were very similar (Figure S14). Thus, the estimated admixture tract distributions do not provide any evidence of Norse Viking admixture.

## Discussion

This study was based on genetic data from more than 10% of the adult present-day Greenlandic population. The availability of this substantial data set has allowed us to provide answers to several previously unresolved questions about the structure and history of the Greenlandic population.

First, our analyses have allowed us to accurately quantify the extent of European ancestry in the present-day Greenlandic population across the island. Our analyses have also allowed us to confirm that there has been a strong male bias among the European ancestors of the Greenlanders. Further, our analyses revealed population structure within the Inuit ancestry component of the population: we roughly observed a genetic subdivision corresponding to the geographic division of Greenland into north, west (including south), and east. Additionally, the observed genetic division also corresponds to the subdivision of the Greenlandic Inuktitut language into three different dialects. The Greenlanders in Qaanaaq predominately speak the Inuktun language (Avanersuarmiutut), the Greenlanders in the west (including the south) speak Kalaallisut, and the Tunumiit in the east predominantly speak Tunumiisut (Tunumiit oraasiat). Interestingly, Qaanaaq in the north and Tasiilaq in the east do not appear to be closer to each other genetically than to other locations, as reported previously.^13^

Second, we found that the Greenlandic population in several ways shows signatures of being a historically isolated and small founder population: it has increased LD, especially in the Inuit ancestral component of the population, and decreased nucleotide diversity. Interestingly, we did not find evidence that the tradition of living in small groups has led to increased rates of inbreeding in Greenlanders relative to Europeans. Furthermore, we found that, unlike other founder populations studied to date, the Greenlandic population is highly genetically differentiated from all large populations, such as Europeans or East Asians, most likely because it has been isolated and affected by bottlenecks in population size and generally small population sizes for a long period of time. However, it should be noted that F_ST_ would presumably be smaller if one compared the Greenlandic population with other Inuit populations, such as Yupik, or Native American and some Siberian populations, which we were not able to do because of a lack of comparable data. These features make the Greenlandic population potentially well suited for genetic mapping of variants associated with disease because increased LD means that fewer SNPs need to be genotyped for obtaining dense genome-wide coverage. Furthermore, the high degree of genetic differentiation, and the strong effect of genetic drift, might suggest that causal variants that are very rare in other populations could segregate at a high frequency in this population. In fact, Moltke et al.^8^ found a variant that is common in Greenland but rare in the rest of the world, which explains more than 10% of all type 2 diabetes cases in Greenland. Thus far, only variants associated with type-2-diabetes-related traits have been mapped in this population. Our results should encourage more association mapping studies on other traits in the Greenlandic population or other historically isolated populations.

Third, we found no support for previous hypotheses suggesting multiple migration events. On the contrary, our results provide multiple lines of evidence that support a single migration wave moving into Northwest Greenland and southward along the west coast and then finally reaching the east coast by passing the south tip of Greenland. It should be noted that we cannot determine from currently available data whether there have been multiple migrations into North Greenland (Qaanaaq) from Canada. The current population in Qaanaaq originated from a migration of central Canadian Inuit in the 17^th^ century, and in 1864 a little group of Inuit from Baffin Island arrived in the Thule district.^43^ This should, however, not change the conclusion of our analyses given that the current population there would be similar to the previous one. However, to be extra careful, we also performed the same analyses by using Upernavik villages instead of Qaanaaq and obtained similar results. What we can conclude is that the data are not compatible with several migrations along the coast of West Greenland or East Greenland, because both Qaanaaq and Upernavik villages form an outgroup to East and West Greenland.

Finally, we found no evidence of interbreeding with Dorset in East Greenland as hypothesized by Helgason et al.^13^ and others. As above, this is based on the argument that the data are compatible with Qaanaaq as a proper outgroup, which we would not expect if the East Greenlanders and the Dorset interbred. We cannot formally exclude that any interbreeding happened, but our data suggest that the Dorset have not contributed much genetic ancestry to the modern East Greenlanders. Likewise, we did not find evidence of interbreeding between Inuit and Norse Vikings. Because the largest Viking settlement was located in Southwest Greenland, we would expect such interbreeding to have left a genetic signature in the individuals in the South villages, and we would not expect it to be present in Qaanaaq. However, we observed no more European ancestry in the South villages than in Qaanaaq. On the contrary, more than half of the individuals were inferred to have no European ancestry at this location, unlike at most of the other locations, including Qaanaaq. Additionally, all individuals with more than 5% European admixture harbored long (>39 cM) ancestral European tracts, which we would expect from very recent admixture. Thus, the most parsimonious explanation is that the admixture in these individuals was caused by very recent admixture and not Norse Viking gene flow. Furthermore, after correcting for admixture proportions, we found that the South villages had no excess of short European ancestry tracts in comparison to Qaanaaq, as we would expect if the Norse Vikings were among the ancestors of the Greenlanders in the South villages and not among the ancestors of the Greenlanders in Qaanaaq. These results do not completely rule out the possibility that the Inuit and the Norse Vikings interbred, but they suggest that the Norse Viking genetic contribution, if any, to the present-day Greenlandic gene pool was minimal.

Several of the above results contradict conclusions drawn from previous studies, presumably because previous studies used small sample sizes and/or a small set of genetic markers. For instance, the hypotheses proposed by Helgason et al.^13^ were based solely on mtDNA and could be explained by incomplete lineage sorting. Other results, such as the lack of Norse Viking admixture, are well in line with previous studies that have found no genetic evidence of such admixture^10^ and thus provide further support for their conclusions.

In conclusion, we have presented the largest genetic data set to date for an Arctic population. Our findings complement the recent ancient-DNA-based study by Raghavan et al.^10^ and provide knowledge about the history of the present-day Greenlandic population.

## Supplemental Data

Document S1. Figures S1–S14 and Tables S1–S3

Document S2. Article plus Supplemental Data
