# Supplementary material for: Uncovering the Genetic History of the Present-Day Greenlandic Population
Source: Am J Hum Genet. 2014 Dec 31;96(1):54–69. doi: 10.1016/j.ajhg.2014.11.012 (PMC4289681; doi:10.1016/j.ajhg.2014.11.012)
Supplement: Document S1. Figures S1–S14 and Tables S1–S3 [file mmc1.pdf]

The American Journal of Human Genetics, Volume *96*

Supplemental Data

Uncovering the Genetic History of the Present-Day Greenlandic Population

Ida Moltke, Matteo Fumagalli, Thorfinn S. Korneliussen, Jacob E. Crawford, Peter Bjerregaard, Marit E. Jørgensen, Niels Grarup, Hans Christian Gulløv, Allan Linneberg, Oluf Pedersen, Torben Hansen, Rasmus Nielsen, and Anders Albrechtsen

Supplemental Data

a b

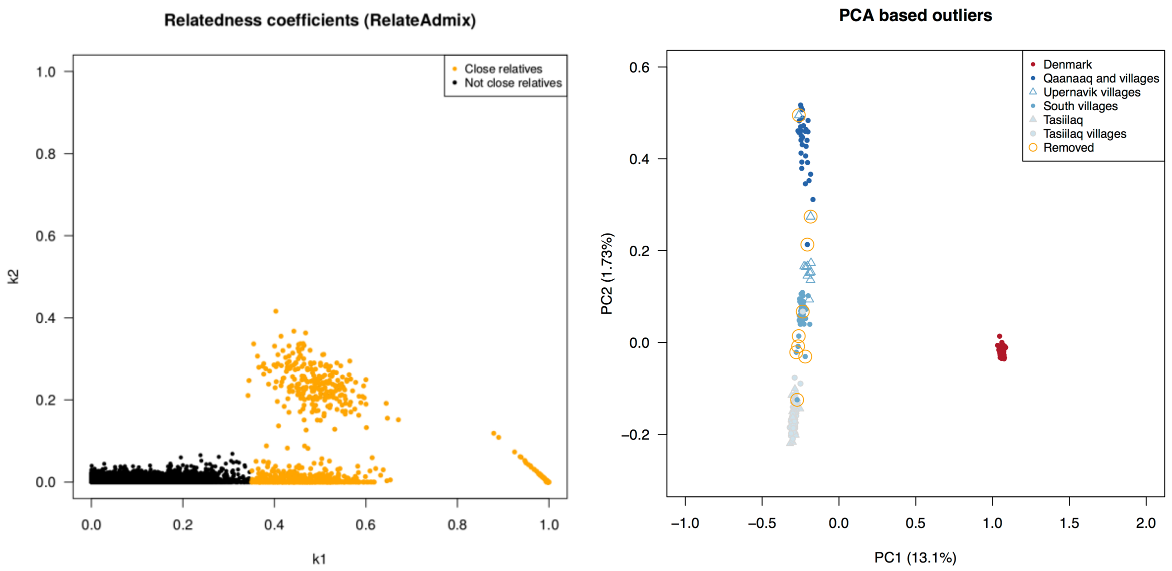

Figure S1. **Analysis results that led to the restricted Greenlandic dataset. a** The estimated relatedness values (k1 and k2) for all pairs of individuals in the subset of individuals from the full dataset that were from Qaanaaq, Upernavik villages, South villages, Tasiilaq or Tasiilaq villages and were estimated to have less than 5% European ancestry. For any pair of individuals k1 is the proportion of their genomes they share one allele Identity-By-Descent (IBD) and k2 is the proportion of their genomes they share two IBD. We removed close relatives based on these relatedness values as follows. First we removed parent-offspring pairs by removing one individual from each pair with k1>0.9. Then we removed full siblings by removing one individual from each remaining pair of individuals with k1>0.25 and k2>0.125. Finally, we removed half sibling/avuncular/grandparent-grandchildren pairs by removing one individual from each remaining pair of individuals with k1>0.35. All pairs with at least one removed individual using this approach are indicated in orange. **b** The first two principal components for the remaining individuals. Nine outliers, and thus potential migrants from other parts of Greenland, were removed (indicated with orange circles).

a **b**

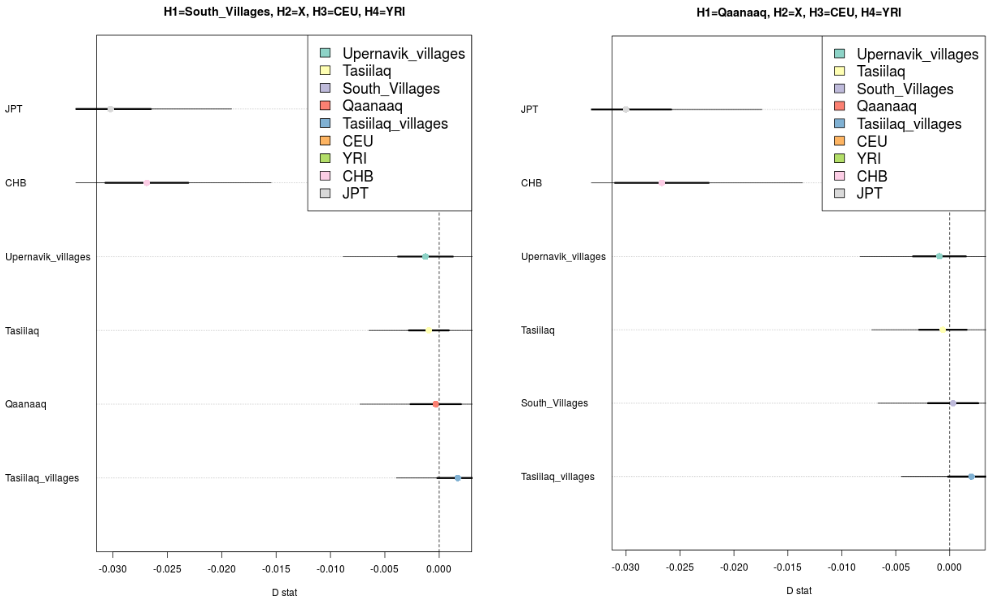

Figure S2. *D*-statistics based on the restricted dataset combined with HapMap samples. The colored points show the point estimates of the *D*-statistic, the thick and thin black lines show 1 standard error and 3 standard errors, respectively. **a** *D*-statistics for topologies of the form (((H1,H2),H3),H4) with H1=South villages, H3=CEU (Europeans), H4=YRI (Africans) and H2 taking different values including Upernavik, Tasiilaq and Qaanaaq. **b** *D*-statistics for topologies of the form (((H1,H2),H3),H4) with H1=Qaanaaq, H3=CEU (Europeans), H4=YRI (Africans) and H2 taking different values including Upernavik, Tasiilaq and South villages. As can be seen none of the *D*-statistics with two Greenlandic locations as ingroups are significantly different from 0, indicating that we see no evidence for admixture between any one of the Greenlandic locations and Europeans.

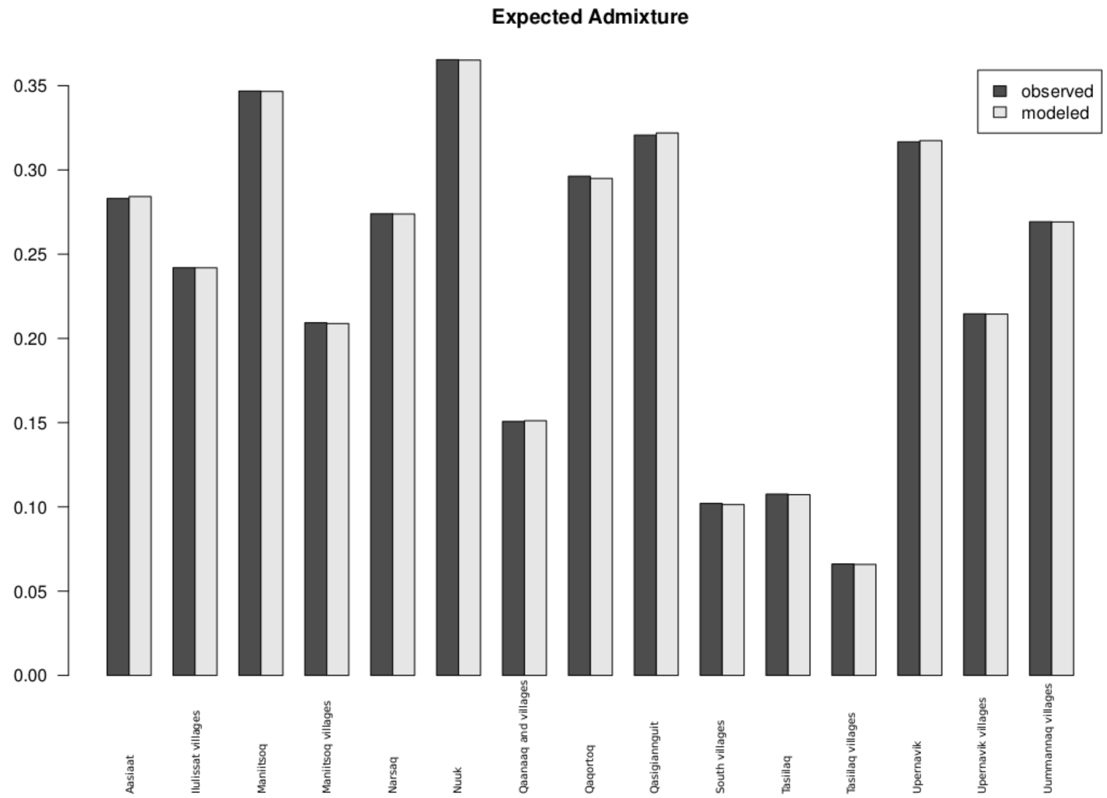

Figure S3. Mean observed and expected European admixture proportions for the full dataset. For each population we show the average observed admixture proportion and the expected value from the modeled distribution used to compute allele frequencies corrected for European admixture.

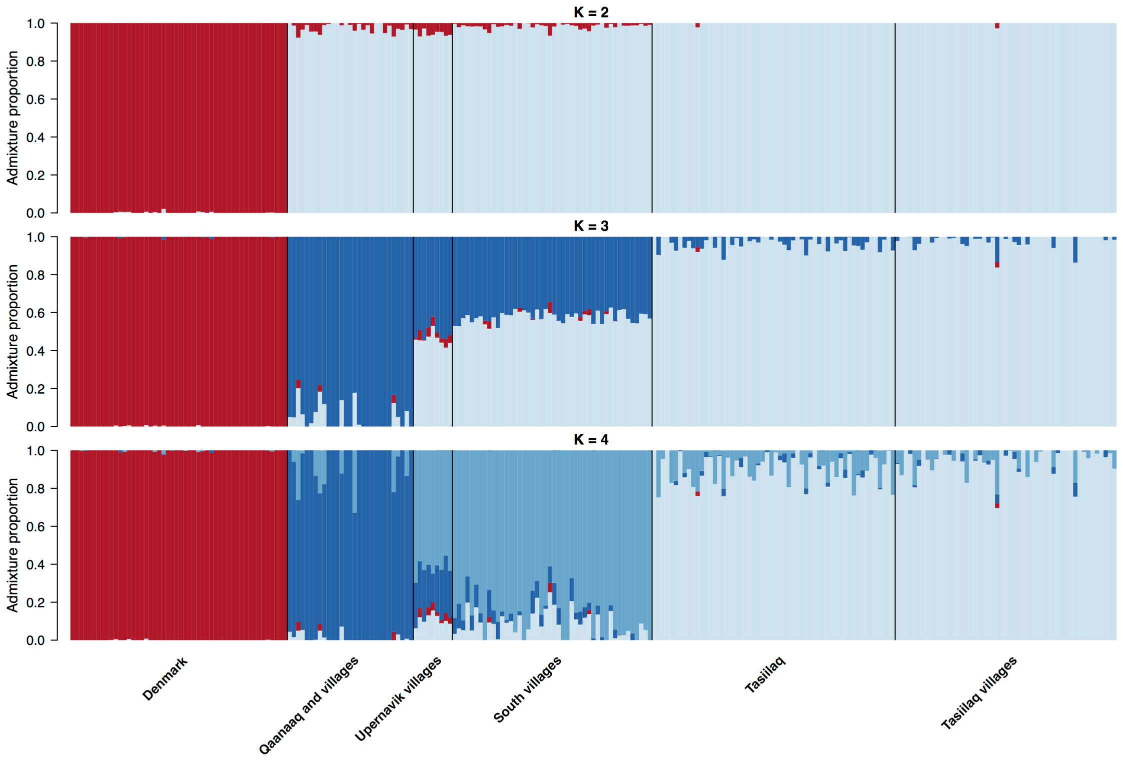

Figure S4. Admixture proportions estimated from the restricted Greenlandic dataset combined with Danish samples. The Greenlandic individuals included in this dataset are not closely related, do not have any recent European ancestry and have not recently migrated within Greenland.

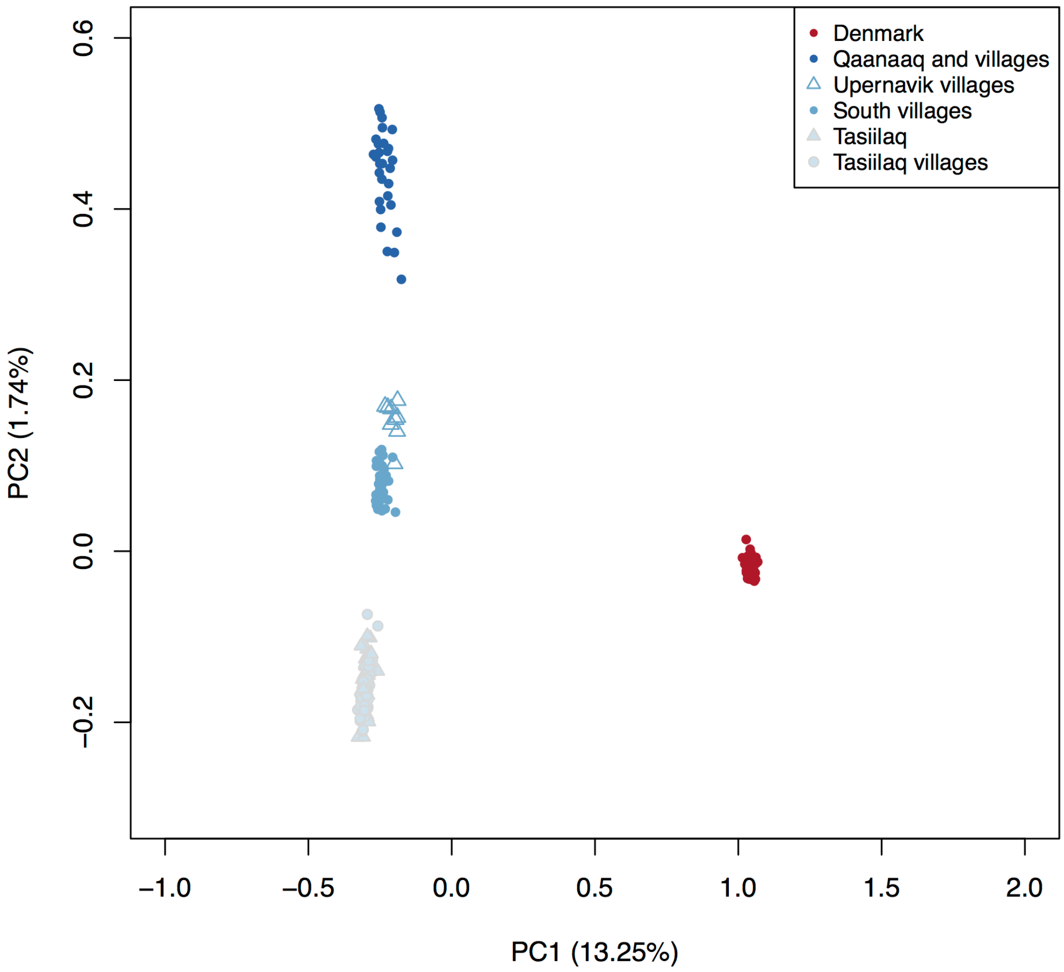

Figure S5. Principal component analysis of the restricted Greenlandic dataset combined with Danish samples. The first two principal components based on a principal component analysis of the genetic covariance matrix of the individuals in the restricted Greenlandic dataset combined with Danish samples, in which the Greenlandic individuals are not closely related, do not have any recent European ancestry and have not recently migrated within Greenland. The estimated percentages of the variation explained by the two principal components are shown in the axis labels. The color scheme is the same as in Figure 1.

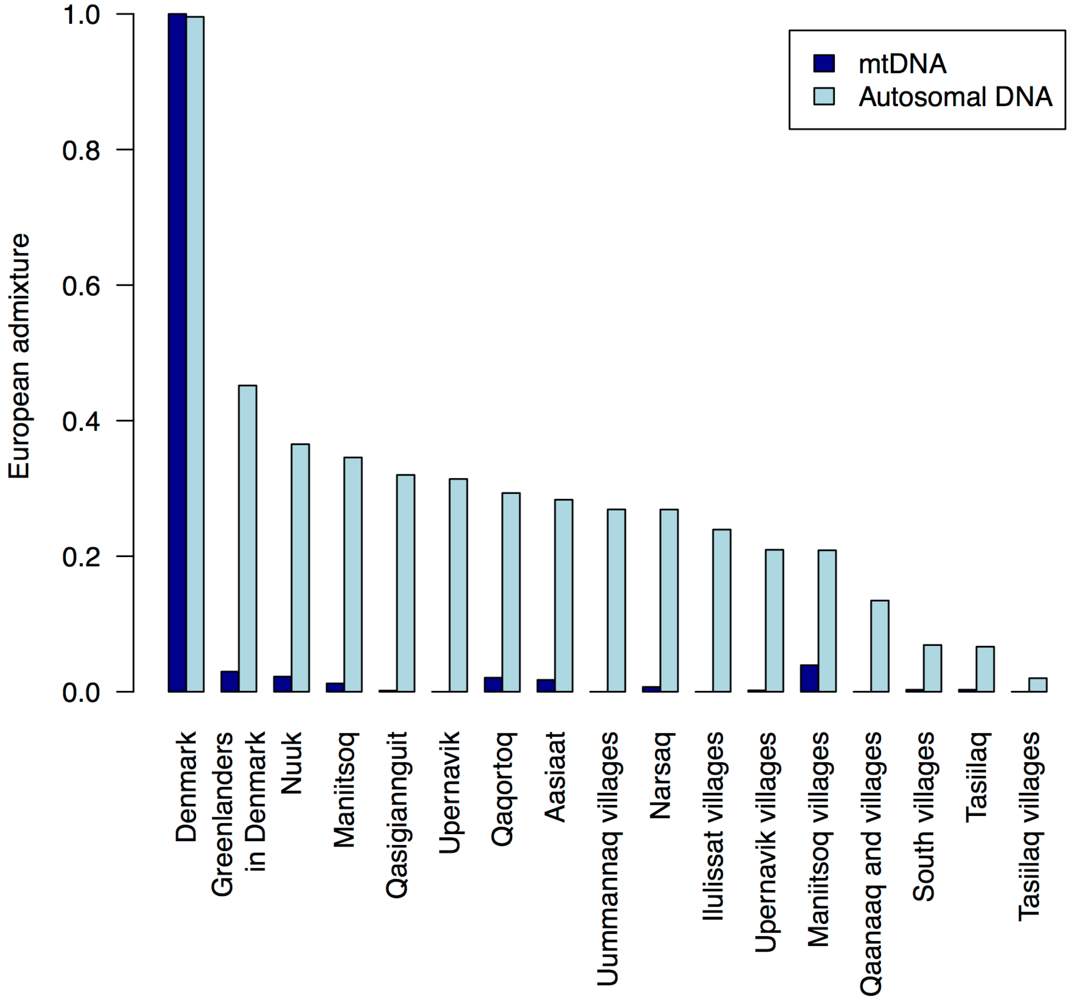

Figure S6. Mean proportion of European ancestry in mtDNA and autosomal DNA for all sampling locations. The proportions were estimated from the full dataset. Among the Greenlanders the autosomal DNA is estimated to have 25.9 times as much European ancestry than the mtDNA.

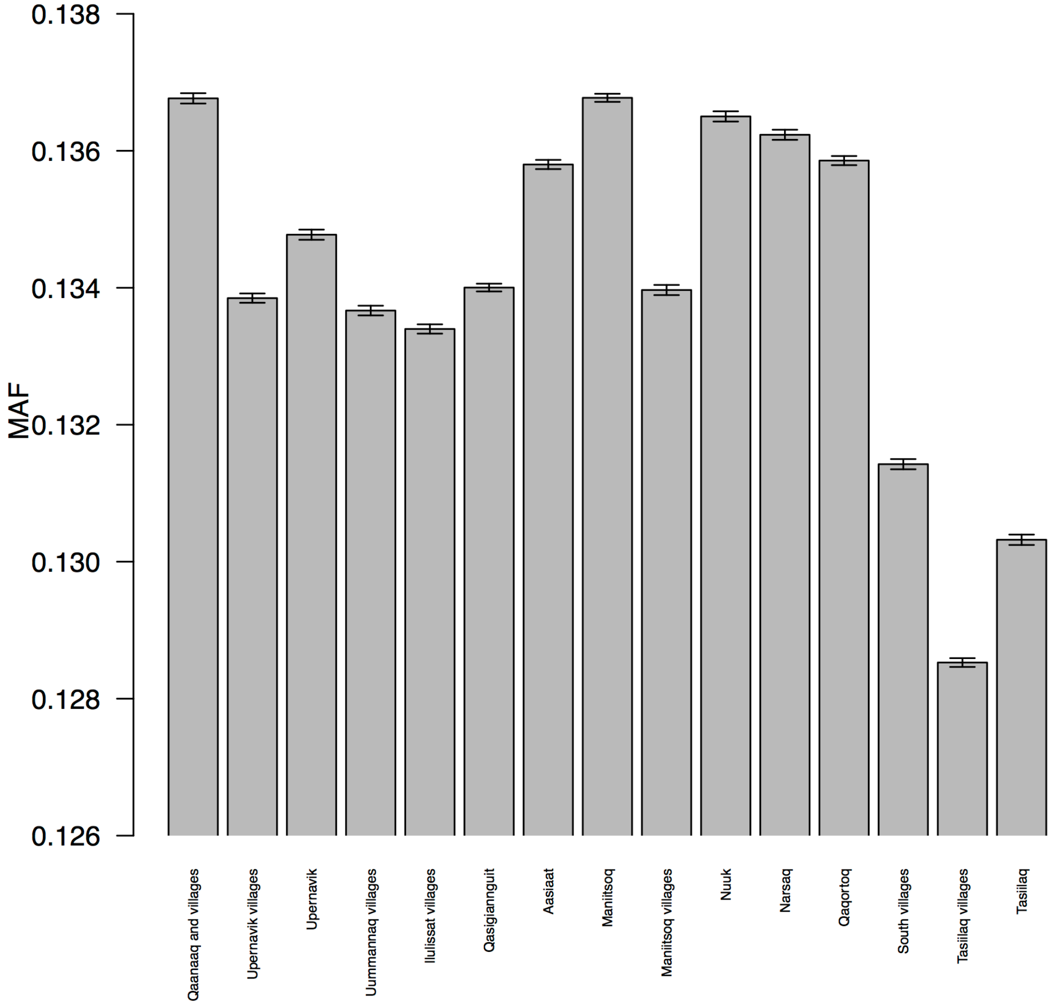

Figure S7. Mean minor allele frequency estimated for all sampling locations in Greenland. The minor allele frequencies (MAFs) were estimated from the full dataset without LD and the Greenlandic allele frequencies were corrected for European admixture. Standard errors achieved using bootstrap are marked with thin black bars around the estimated means.

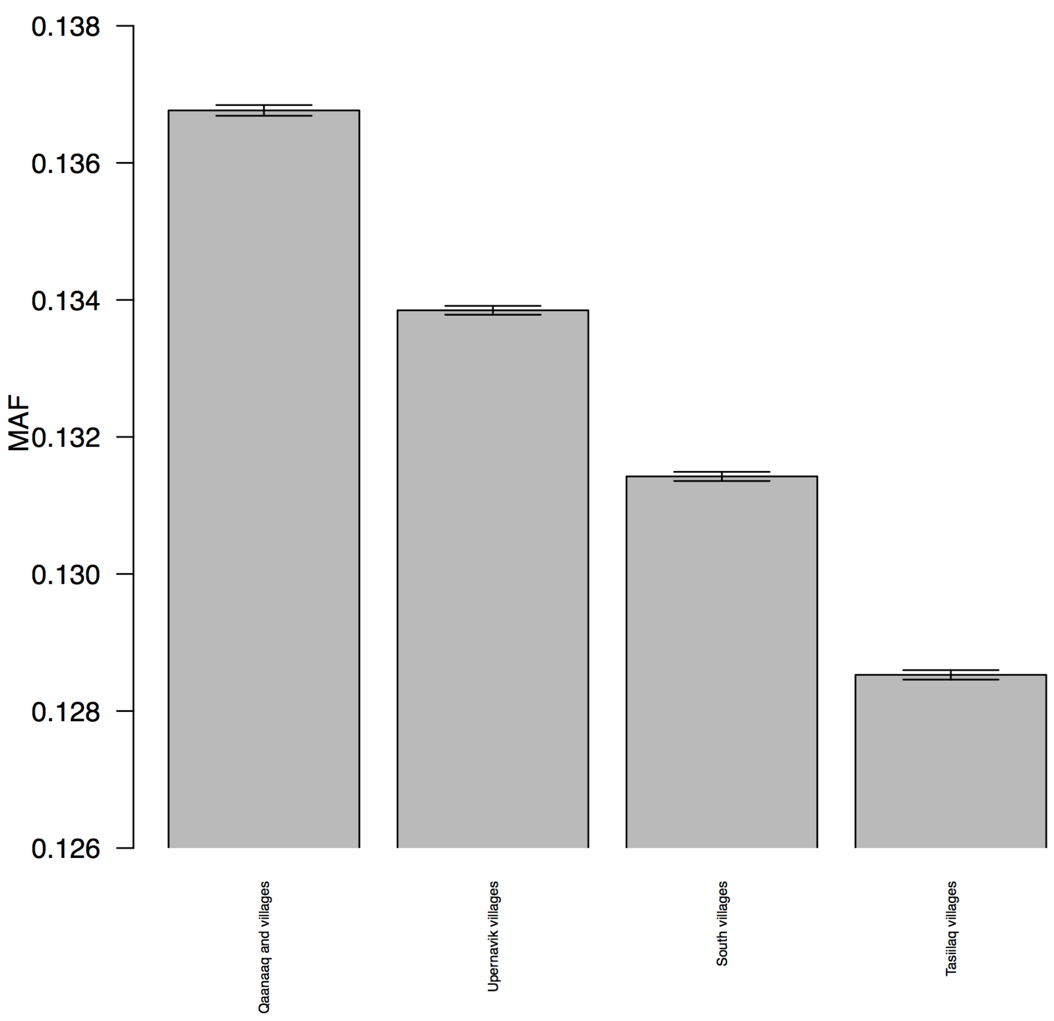

Figure S8. Mean minor allele frequency estimated in four different sampling locations. The locations are: Qaanaaq (North), Upernavik (West), South villages (South) and Tasiilaq villages (East). The minor allele frequencies (MAFs) were estimated from the full dataset without LD and were corrected for European admixture. Standard errors achieved using bootstrap are marked with thin black bars around the estimated means.

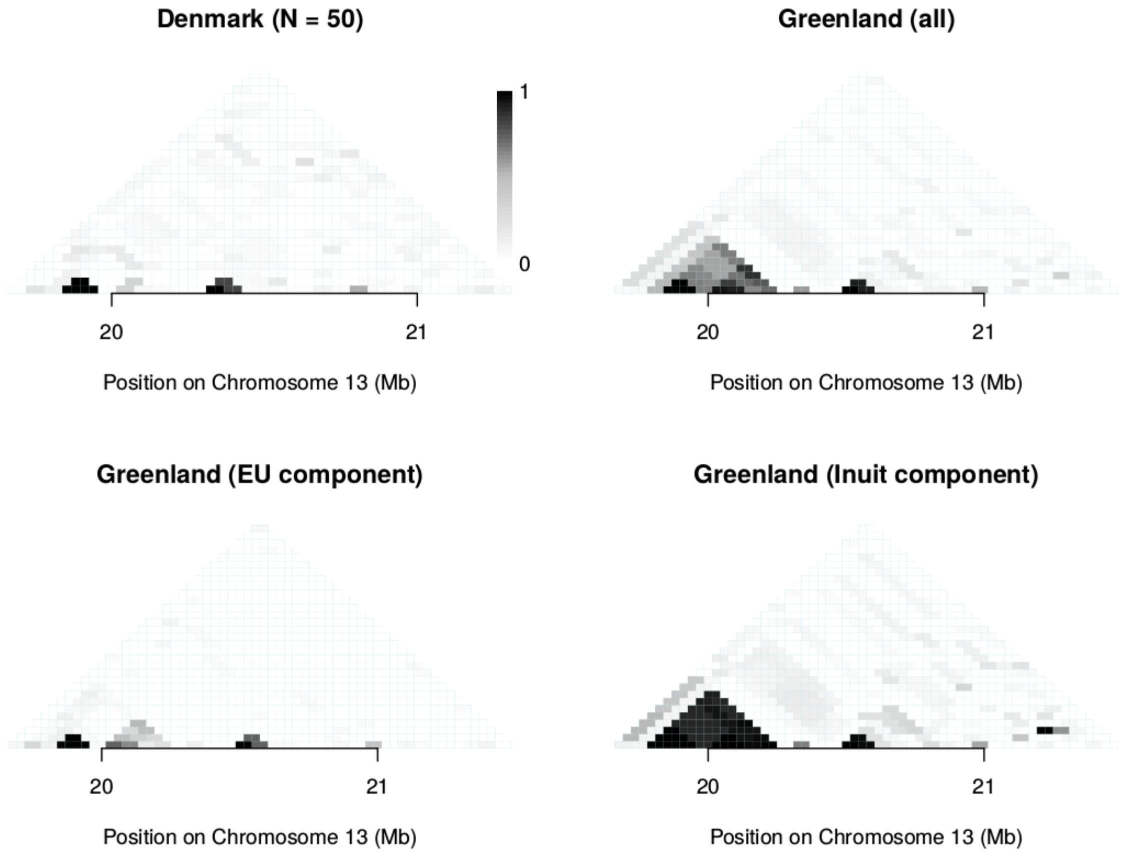

Figure S9. Haplotype blocks in different populations. Pairwise linkage disequilibrium (LD) estimates measured by *r*² shown in tile diagrams. The tile diagrams in the top row show the LD estimated in the 50 Danish individuals and in all of the Greenlandic individuals (all). The diagrams in the bottom row show LD estimated for the ancestral European and ancestral Inuit part of the Greenlandic individuals' ancestry (EU component and Inuit component). Chromosome 13 was randomly chosen and only the first megabases (Mbs) with data are shown. This region is an example of the difference in haplotype block sizes between Europeans and Inuit.

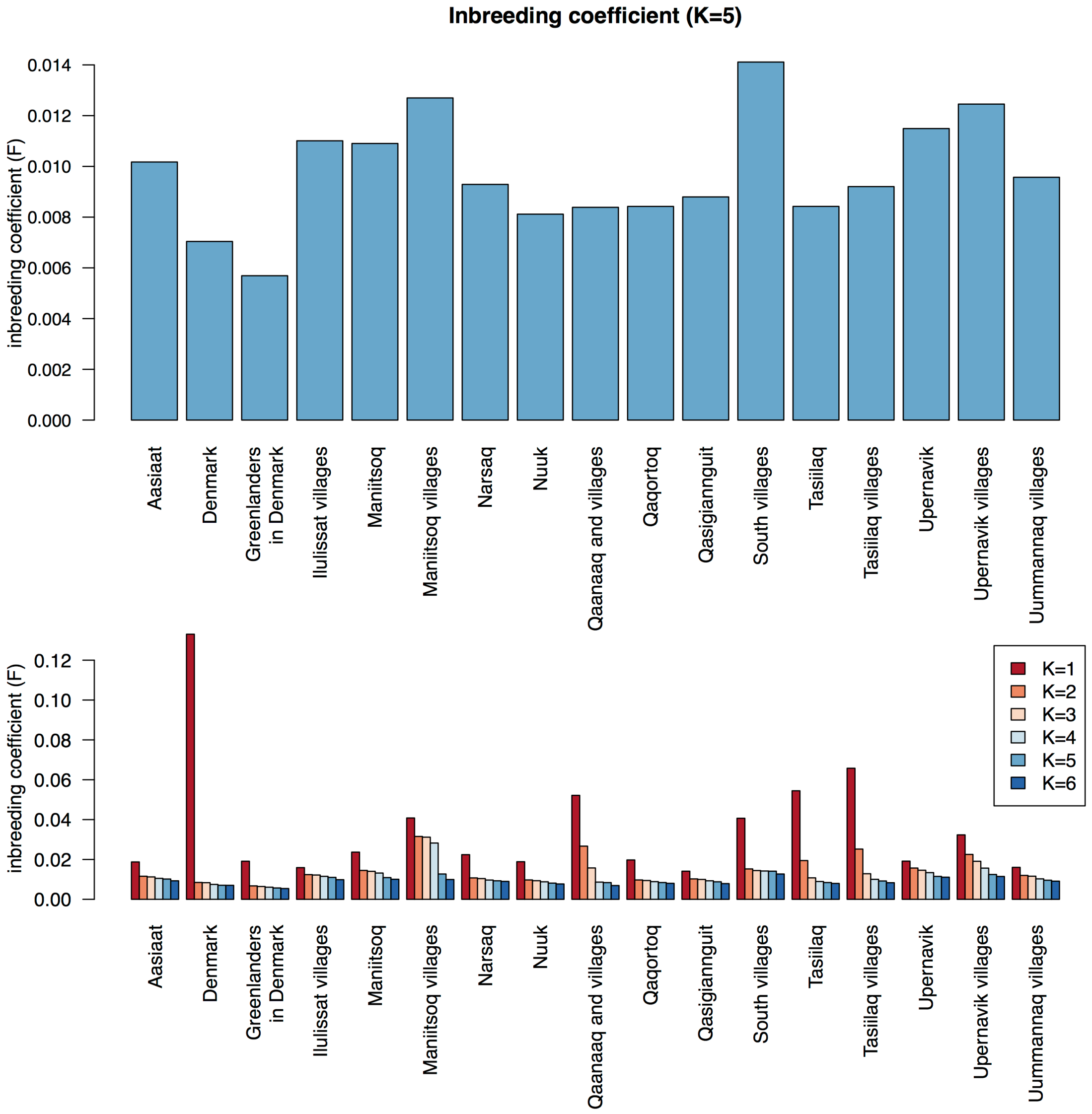

Figure S10. Estimated mean inbreeding coefficients. The inbreeding coefficients were estimated for different locations in Greenland, for Danes and for Greenlanders living in Denmark. The estimates were based on analyzing all the individuals in the study (the full dataset) and are corrected for admixture assuming different number of ancestral populations (K=1-6). The top plot shows the inbreeding estimated after correcting for admixture assuming 5 ancestral populations. The bottom plot shows the results for different number of assumed ancestral populations. As can be seen from the bottom plot increasing K by one changes the estimates markedly for all K<5, whereas the estimates change very little when increasing K from 5 to 6. This observation is the reason why the results for K=5 are shown in detail in the top plot.

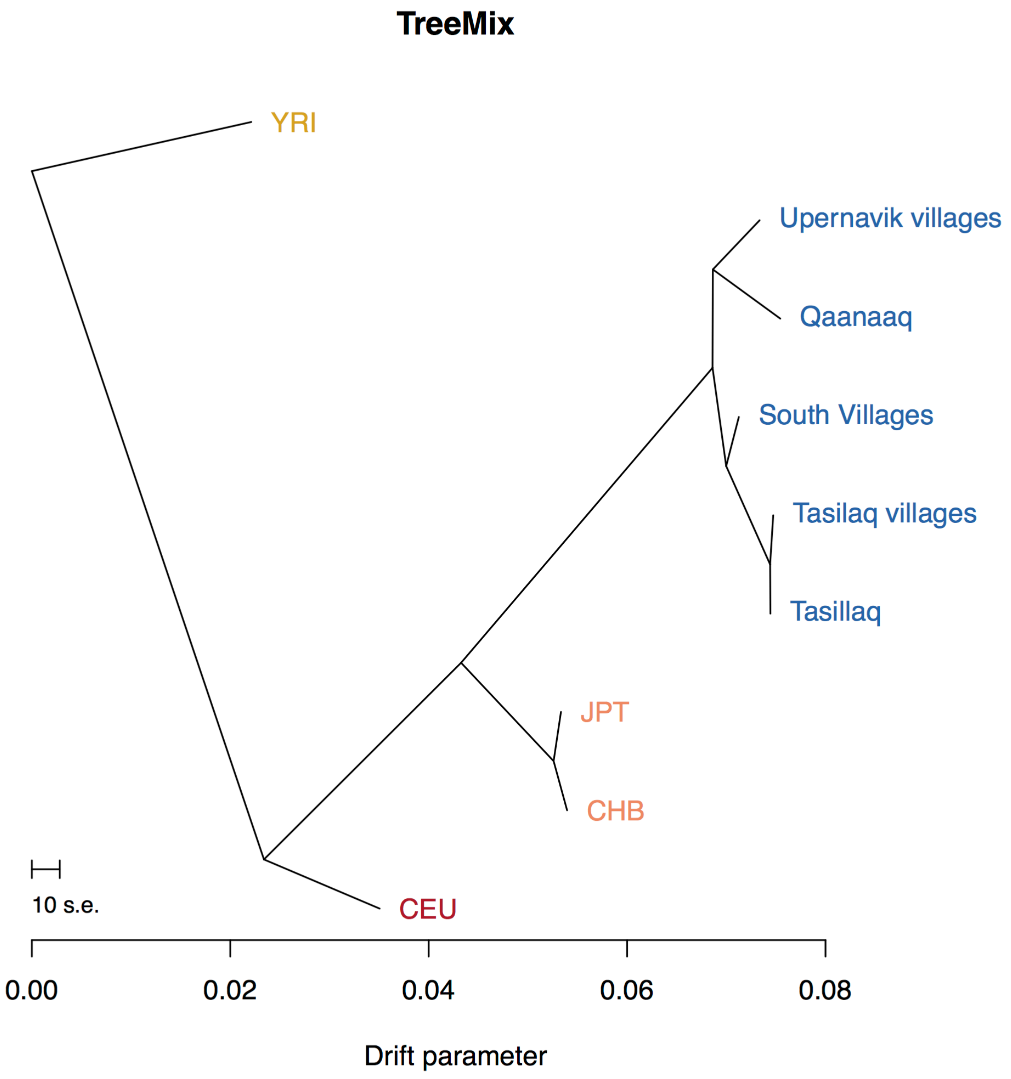

Figure S11. TreeMix results for the restricted Greenlandic dataset combined with HapMap samples. The results of running TreeMix assuming 0 admixture events.

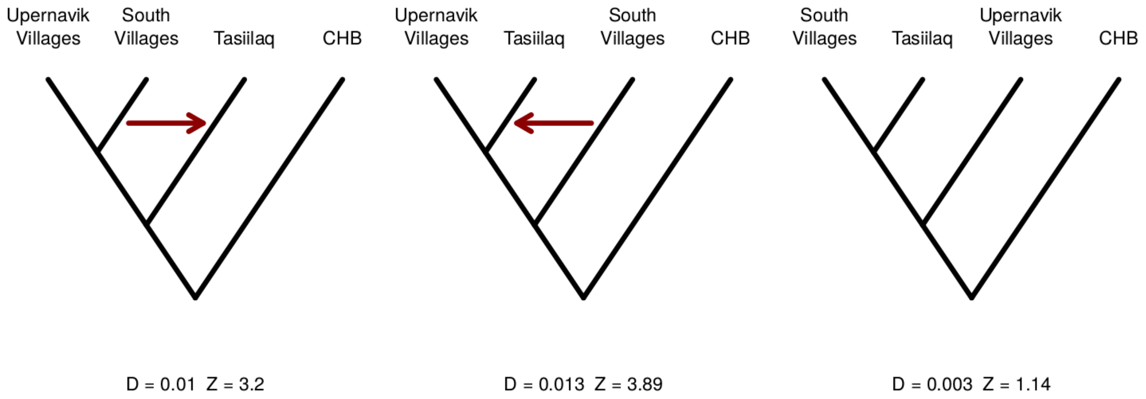

Figure S12. *D*-statistics for different possible topologies including Upernavik villages. The *D*-statistics were estimated from the restricted Greenlandic dataset combined with HapMap samples. The Han Chinese (CHB) HapMap samples are used as outgroup. This figure is similar to figure 8, but includes Upernavik villages instead of Qaanaaq. The conclusions of the analyses are the same.

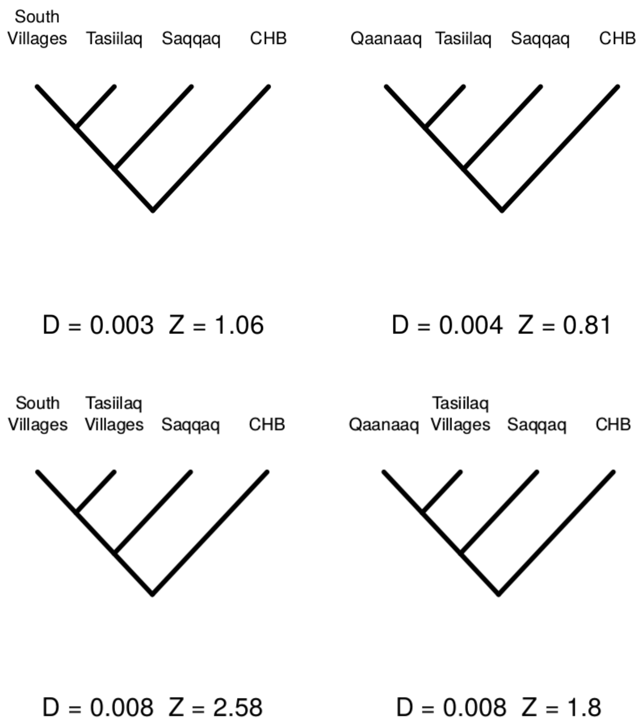

Figure S13. *D*-statistics estimated to test for possible Dorset admixture in East Greenland using an ancient Saqqaq genome as a representative for the Dorset. When merging the sequencing data from the Saqqaq genome and the SNP chip data from this study A/T and C/G sites were removed to avoid any potential errors due to strand ambiguity.

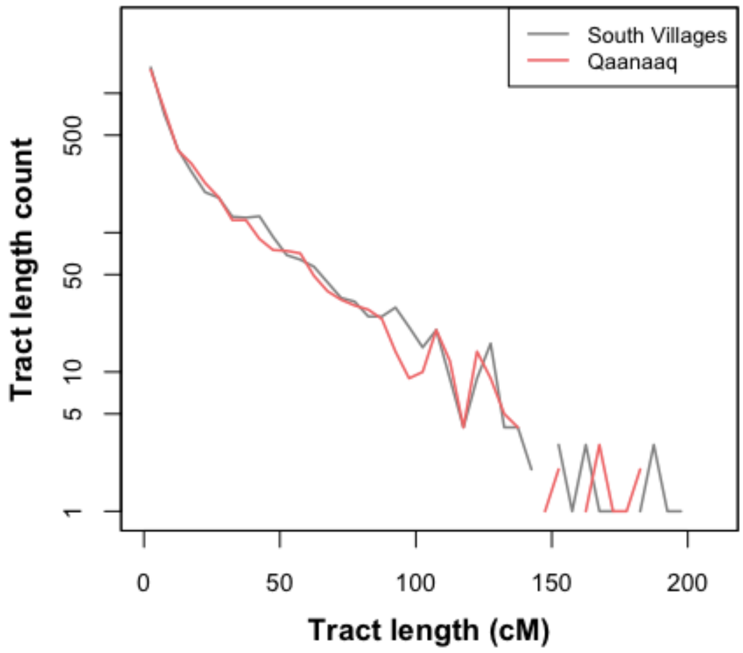

Figure S14. The distribution of European admixture tract lengths in admixed individuals from the South villages and Qaanaaq. The distributions were inferred from 40 individuals from South villages and 40 individuals from Qaanaaq. They are highly correlated and do not provide support for Norse admixture in the South villages. Local ancestry was estimated using RFMix with Danish (European) and unadmixed South villages (Inuit) reference populations. Tract length distributions were discretized into 5cM bins after normalizing population-level admixture proportions. Note that the y-axis is shown on a logarithmic scale. The lines are truncated in bins with tract length sizes that are not observed.

	CEU	CHB	JPT	Qaanaaq	South villages	Tasiilaq villages	Tasiillaq	Upernavik villages	YRI
CEU	0	0.12	0.12	0.17	0.16	0.17	0.17	0.16	0.15
CHB	0.12	0	0.01	0.13	0.12	0.13	0.13	0.12	0.19
JPT	0.12	0.01	0	0.12	0.11	0.12	0.13	0.12	0.19
Qaanaaq	0.17	0.13	0.12	0	0.04	0.04	0.04	0.04	0.25
South villages	0.16	0.12	0.11	0.04	0	0.02	0.02	0.03	0.24
Tasiilaq villages	0.17	0.13	0.12	0.04	0.02	0	0	0.04	0.26
Tasiilaq	0.17	0.13	0.13	0.04	0.02	0	0	0.04	0.26
Upernavik villages	0.16	0.12	0.12	0.04	0.03	0.04	0.04	0	0.24
YRI	0.15	0.19	0.19	0.25	0.24	0.26	0.26	0.24	0

Table S1. Pairwise F_{ST} estimated from the restricted Greenlandic dataset combined with HapMap samples. The estimates were obtained using the Weir and Cockerham estimator. Since the restricted dataset was used the Greenlandic individuals included in this analysis are not closely related, do not have any recent European ancestry and have not recently migrated within Greenland.

	Denmark	CEU*	JPT	CHB	Qaanaaq	South villages	Tasiilaq villages	Tasiilaq	Upernavik villages
N	50	60	44	42	29	46	51	56	9
Mt1736 frequency	1	1	0.95	0.88	0	0	0	0	0

Table S2. Frequency of mtDNA mt1736 in unadmixed individuals. N is the number of individuals with non-missing genotypes. CEU are European individuals from HapMap and JPT+CHB are Japanese and Chinese individuals from HapMap. Note that the CEU data (marked with a *) are for 30 trios, i.e. 60 unrelated individuals.

	CEU	CHB	JPT	YRI	Greenland
Variability	0.221%	0.209%	0.206%	0.341%	0.152%
Avg. MAF*	17.5%	17.7%	17.8%	14.6%	21.1%
Avg. MAF	0.4454%	0.4448%	0.4447%	0.4508%	0.4429%
Avg DAF	28.4%	29.1%	29.3%	21.8%	35.5%
$\theta\pi$	0.00056	0.00053	0.00053	0.00073	0.00046
F_{ST} with Greenland	0.1635	0.1235	0.1203	0.2249	-

Table S3. **Summary information from sequencing data**. The information includes the fraction of sites that are polymorphic (variability), mean minor allele frequency among polymorphic sites (avg. MAF*), mean minor allele frequency among all sites (avg. MAF), mean derived allele frequency (avg. DAF), $\Theta\pi$ and F_{ST} with Greenland. All the information is based on the estimated site frequency spectra (SFSs) for 18 Greenlanders and 18 individuals from each of the 4 original HapMap populations. Pairwise F_{ST} with Greenland was estimated from 2D SFSs. The Greenlanders were exome sequenced while the HapMap populations were sequenced as part of the 1000 genomes project. Only the 75Mb extended target regions defined by Agilent SureSelect were used for all 5 populations.
